# Myelodysplastic neoplasm-associated U2AF1 mutations induce host defense defects by compromising neutrophil chemotaxis

**DOI:** 10.1038/s41375-023-02007-7

**Published:** 2023-08-17

**Authors:** Natalia J. Gurule, Kenneth C. Malcolm, Chelsea Harris, Jennifer R. Knapp, Brian P. O’Connor, Jazalle McClendon, William J. Janssen, Frank Fang Yao Lee, Caitlin Price, Jackson Osaghae-Nosa, Emily A. Wheeler, Christine M. McMahon, Eric M. Pietras, Daniel A. Pollyea, Scott Alper

**Affiliations:** 1 Department of Immunology and Genomic Medicine, National Jewish Health, Denver, CO USA; 2 Center for Genes, Environment and Health, National Jewish Health, Denver, CO USA; 3 Department of Immunology and Microbiology, University of Colorado School of Medicine, Anschutz, CO USA; 4 Department of Medicine, National Jewish Health, Denver, CO USA; 5 Department of Medicine, University of Colorado, Aurora, CO USA

**Keywords:** Myelodysplastic syndrome, Immunological disorders

## Abstract

Myelodysplastic neoplasm (MDS) is a hematopoietic stem cell disorder that may evolve into acute myeloid leukemia. Fatal infection is among the most common cause of death in MDS patients, likely due to myeloid cell cytopenia and dysfunction in these patients. Mutations in genes that encode components of the spliceosome represent the most common class of somatically acquired mutations in MDS patients. To determine the molecular underpinnings of the host defense defects in MDS patients, we investigated the MDS-associated spliceosome mutation U2AF1-S34F using a transgenic mouse model that expresses this mutant gene. We found that U2AF1-S34F causes a profound host defense defect in these mice, likely by inducing a significant neutrophil chemotaxis defect. Studies in human neutrophils suggest that this effect of U2AF1-S34F likely extends to MDS patients as well. RNA-seq analysis suggests that the expression of multiple genes that mediate cell migration are affected by this spliceosome mutation and therefore are likely drivers of this neutrophil dysfunction.

## Introduction

Myelodysplastic neoplasm (MDS) is a hematopoietic stem cell disorder characterized by defects in myeloid cell differentiation, production of dysplastic blood cells, and risk of evolution to acute myeloid leukemia (AML) [1]. Approximately 10,000–40,000 patients are newly diagnosed with MDS in the United States annually [2–4]. MDS occurs primarily in elderly individuals [5] and, like AML, has a poor prognosis, with only a 35% 3-year survival rate [6]. In MDS patients, defects in myeloid stem cell differentiation can induce anemia due to erythrocyte defects, hemorrhage due to platelet disorders, and increased infection risk due to neutropenia or neutrophil dysfunction. Fatal infection and infectious complications are among the most common cause of death in MDS patients, accounting for between 18% and 53% of all deaths in MDS patients [7–10]. Gram negative and Gram positive bacterial infections are fairly common in MDS patients; while viral and fungal infections also occur, these are rarer.

Many patients with MDS are neutropenic, which accounts for some but not all of the increased infection risk in these patients [11–16]. Even when neutrophils are produced in patients with MDS, these neutrophils exhibit a range of functional defects, and this neutrophil dysfunction also likely accounts for the increased susceptibility to infection in patients with MDS [11–16].

What drives this neutrophil dysfunction and the resulting increased infection risk in MDS patients is still unclear. Recent next-generation sequencing studies have identified many recurrent mutations in MDS patients [17]. The most common class of genes that are somatically mutated in MDS are those that regulate pre-mRNA splicing, with roughly half of all MDS patients having a mutation in the splicing machinery [18]. The removal of introns from pre-mRNA is driven by the spliceosome, a large ribonucleoprotein complex [19]. Three spliceosome genes, U2AF1, SF3B1, and SRSF2 are frequently somatically mutated in MDS patients [20–22]. These spliceosome mutations act as gain of function or neomorphic mutations [20–22] that induce changes in gene expression and pre-mRNA splicing on a genomic scale [20–22]. Mice engineered to express these MDS-associated spliceosome gene mutations exhibit hematopoietic defects [23, 24] suggesting that these spliceosome mutations are driver mutations that contribute to disease pathogenesis.

In particular, mice engineered to express the common U2AF1-S34F mutation exhibit hematopoietic defects [25, 26]. Expression of human U2AF1-S34F in mice induced cytopenias in B cells and monocytes and affected common myeloid progenitor production [25]. Inhibition of U2AF1 or other MDS-associated splicesome mutations weakens inflammation by altering splicing in TLR signaling pathways [27, 28]. In contrast, the neomorphic MDS-associated U2AF1-S34F mutation and other spliceosome mutations enhance pro-inflammatory signaling pathways [28–33]. The effects of altered innate immune signaling induced by MDS-associated spliceosome mutations on host defense have not been investigated previously even though MDS patients exhibit substantial immunodeficiency.

Here we investigate the effect of the MDS-associated U2AF1-S34F mutation on host defense using mice engineered to express human U2AF1-S34F and using peripheral blood samples from patients with MDS. We find that mice expressing U2AF1-S34F are profoundly immunodeficient, rapidly succumbing to *E. coli* infection. This is likely due to neutrophil chemotaxis defects induced by the U2AF1-S34F mutation. Multiple gene expression changes in neutrophils from U2AF1-S34F mice may drive this neutrophil dysfunction. Studies using neutrophils from MDS patients suggest that U2AF1 mutation weakens neutrophil chemotaxis ability in humans as well.

## Materials and methods

### U2AF1 mice

All mouse studies were approved by the National Jewish Health Institutional Animal Care and Use Committee with the protocol number AS2801-05-24. Mice engineered to inducibly express wild type U2AF1 (U2AF1-wt, rtTA) or the U2AF1-S34F mutant variant (U2AF1-S34F, rtTA) have been described [25]. These mice express human U2AF1 transgenes; mouse and human U2AF1 differ in only one amino acid and are >99% identical. Transgenic mice were bred with C57BL/6J-129 wild type mice to generate heterozygous U2AF1-S34F and U2AF1-wt mice following the breeding scheme outlined in [25]. To induce U2AF1 transgene expression, 8–12 week old male and female mice were injected via intraperitoneal injection with 25 mg/kg 9-tert-Butyl Doxycyline (9-TB) in PBS daily for four consecutive days. All experiments were then initiated on the fifth day.

See online Supplementary Methods for all other Methods.

## Results

### Mice expressing U2AF1-S34F are susceptible to infection

To assess the effect of the MDS-associated U2AF1-S34F mutation on host defense, we analyzed mice engineered to express U2AF1-S34F using a rtTA doxycycline-inducible system (rtTA, U2AF1-S34F) compared to control mice engineered to express a wild type U2AF1 transgene (rtTA, U2AF1-wt) [25]. Because doxycycline has antimicrobial properties, we induced transgene expression using a doxycycline analog (9-tert-Butyl Doxycycline HCl or 9-TB) that was previously reported to not exhibit antimicrobial activity [34], and which we confirmed did not kill *E. coli* (Supplementary Fig. 1).

**Fig. 1:**
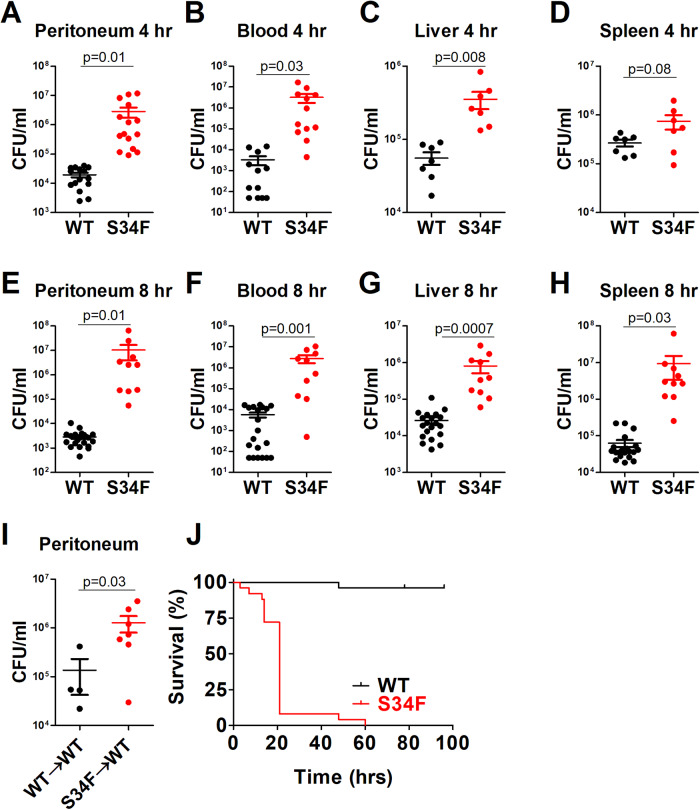
Mice expressing U2AF1-S34F are unable to control an *E. coli* infection. U2AF1-S34F, rtTA mice and control U2AF1-wt, rtTA mice were injected daily for four days with 9-TB via intraperitoneal injection. The mice were subsequently infected with 10^8^ CFU of *E. coli* strain H9049 via intraperitoneal injection. Four (**A**–**D**) or eight (**E**–**H**) hours later, the mice were humanely euthanized, and viable bacteria were quantitated by counting colony forming units (CFUs) in peritoneal lavage, blood, liver, and spleen. In all cases, U2AF1-S34F mice had significantly higher CFU counts than the control U2AF1-wt mice. CFU counts in some blood samples from U2AF1-wt mice were not detected on the lowest dilution plate, and are graphed as the maximum theoretical CFU count. **I** To confirm that hematopoietic expression of U2AF1-S34F was inducing this host defense defect, bone marrow in wild type recipient mice was ablated with lethal irradiation, and donor marrow from either U2AF1-S34F, rtTA mice or U2AF1-wt, rtTA mice was implanted in the recipients. Six weeks later, transgene expression was induced by four daily injections of 9-TB. Subsequently, the mice were infected via intraperitoneal injection with 10^8^ CFU of *E. coli* strain H9049; CFU counts were determined 4 h later. **J** U2AF1-S34F, rtTA and U2AF1-wt, rtTA mice were injected with 9-TB daily for 4 days and were then infected via intraperitoneal injection with 10^8^ CFU of *E. coli* strain H9049. Mouse morbidity was then monitored. *N* = 26 in each group, *p* < 0.0001. In this figure and all subsequent figures, WT (black dots/lines) indicate U2AF1-wt, rtTA mice; S34F (red dots/lines) indicates U2AF1-S34F mice.

Mice heterozygous for the U2AF1-S34F transgene and control mice heterozygous for U2AF1-wt were injected daily for four days via intraperitoneal injection with 9-TB, which induced moderate overexpression of the U2AF1 transgenes similar to expression induced by including doxycycline in the mouse chow [28]. One day after the last 9-TB injection, mice were injected via intraperitoneal injection with 10^8^ CFU *E. coli* strain H9049, a human patient isolate of *E. coli*. Four or eight hours after infection, the mice were humanely euthanized, and viable bacteria in peritoneal lavage, blood, liver, and spleen were assessed by counting colony forming units (CFUs) (Fig. 1A–H). In all cases, significantly more viable bacteria were recovered from the mice expressing the U2AF1-S34F transgene compared to mice expressing U2AF1-wt.

The rtTA, U2AF1-S34F mice express the U2AF1 transgene in most tissues. To confirm that U2AF1-S34F was inducing host defense defects due to its expression in the hematopoietic system, we lethally irradiated wild type recipient mice and then transferred marrow from either rtTA, U2AF1-S34F mice or rtTA, U2AF1-wt mice into the irradiated recipients. Six weeks after marrow transfer, the recipient mice were injected daily for four days with 9-TB, and then the mice were infected with *E. coli* strain H9049. Mice expressing U2AF1-S34F in the hematopoietic system, but not mice expressing U2AF1-wt, exhibited higher viable bacterial counts in peritoneal lavage after infection (Fig. 1I).

To determine the effect of U2AF1-S34F on host defense, we also monitored survival of U2AF1-S34F mice and U2AF1-wt control mice after infection with *E. coli*. While almost all wild type mice were able to survive the infection, U2AF1-S34F mice exhibited significant morbidity, with almost all mice succumbing to infection within 24 h (Fig. 1J). Thus, mice expressing U2AF1-S34F exhibited significant host defense defects as assessed by bacterial CFU counts and mouse morbidity.

### Neutrophils in mice expressing U2AF1-S34F fail to migrate to the infection site

To determine why U2AF1-S34F mice succumbed to *E. coli* infection, we monitored the production of the pro-inflammatory cytokines TNFα and IL-6 in the peritoneum and blood after peritoneal *E. coli* infection. Mice expressing U2AF1-S34F produced as much or more pro-inflammatory cytokines in their peritoneum and blood than did mice expressing U2AF1-wt (Supplementary Fig. 2A–H), indicating that despite their host defense deficiency, U2AF1-S34F mice were still capable of detecting the presence of the pathogen.

**Fig. 2:**
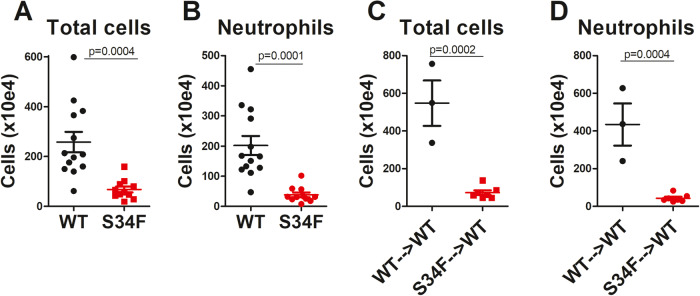
Neutrophils fail to migrate to the infection site in U2AF1-S34F mice. **A**, **B** U2AF1-S34F, rtTA mice and U2AF1-wt, rtTA mice were injected daily with 9-TB for 4 days and then were infected via intraperitoneal injection with 10^8^ CFU *E. coli* strain H9049. Six hours later, total cells in peritoneal lavage were counted with a hemocytometer. Cell differentials were analyzed by staining cytospin slides; total neutrophil counts were calculated by multiplying the fraction of total cell that were neutrophils. The remaining cells were all monocytes/macrophages. **C**, **D** Bone marrow was ablated in wild type recipient mice, and then marrow from either U2AF1-S34F, rtTA or U2AF1-wt, rtTA donor mice was injected via tail vein injection. Six weeks after marrow engraftment, the mice were injected daily for four days with 9-TB and were then infected with *E. coli* via intraperitoneal injection. Four hours after infection, total cell counts and neutrophil counts in the peritoneum were analyzed as described in (**A**) and (**B**).

We also examined recruitment of inflammatory cells to the peritoneum after peritoneal *E. coli* infection. Intraperitoneal (I.P.) infection with *E. coli* induced a robust inflammatory cell infiltration composed of neutrophils and monocytes/macrophages in mice expressing U2AF1-wt. In contrast, neutrophils in mice expressing U2AF1-S34F largely failed to migrate to the site of infection (Fig. 2A, B). Bone marrow chimera studies performed as described above confirmed that this neutrophil chemotaxis defect was caused by U2AF1-S34F expression in the hematopoietic system. I.P. *E. coli* infection induced robust inflammatory cell infiltration into the peritoneum in mice receiving wild type U2AF1 marrow; in contrast, mice that received U2AF1-S34F marrow had a significant defect in neutrophil recruitment to the peritoneum (Fig. 2C, D).

### Neutrophils from U2AF1-S34F mice exhibit an intrinsic migration defect

There are several possibilities that could explain why neutrophils fail to migrate to the infection site in U2AF1-S34F mice following peritoneal infection. These include: (1) lack of neutrophil production in the U2AF1-S34F mice, (2) lack of signals needed for neutrophil recruitment in U2AF1-S34F mice, or (3) an intrinsic migration defect in neutrophils in U2AF1-S34F mice. We tested each of these three possibilities in turn as outlined below.

**Fig. 3:**
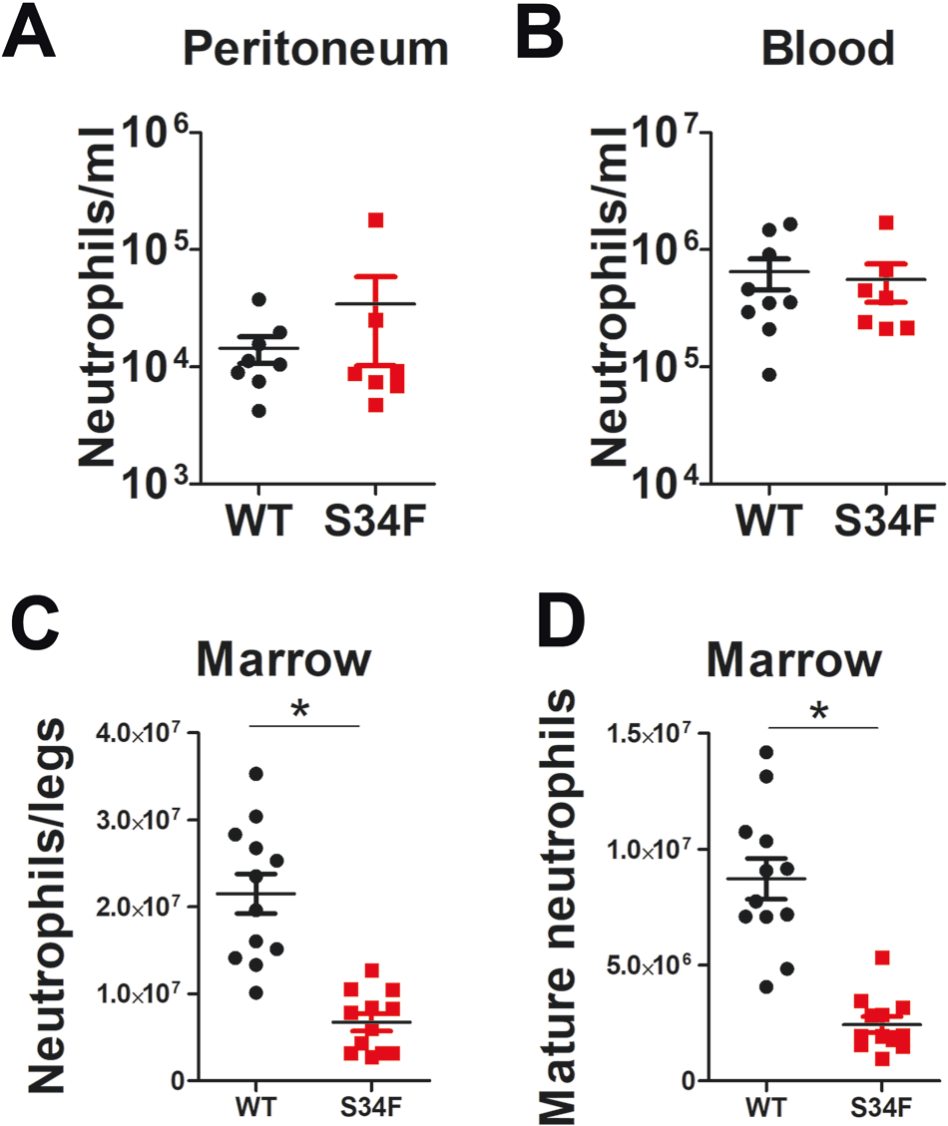
Neutrophils are present in U2AF1-S34F mice. U2AF1-S34F, rtTA and U2AF1-wt, rtTA mice were injected daily for 4 days with 9-TB. The mice were subsequently euthanized, and neutrophil counts in peritoneal lavage, blood, and bone marrow were assessed by flow cytometry. Neutrophil counts in lavage and blood are reported as total neutrophils per ml lavage fluid or blood, respectively. Neutrophils in bone marrow are reported as total neutrophils or total mature neutrophils per mouse, quantifying four leg bones (femurs and tibias) in each mouse.

First, we quantitated the basal number of neutrophils present in uninfected mice, and found that neutrophil counts were similar in peritoneum and blood in mice expressing U2AF1-S34F or U2AF1-wt (Fig. 3A, B). In the bone marrow, while neutrophils were present, there was a significant reduction in the total number of neutrophils in the U2AF1-S34F mice (Fig. 3C) and a comparable decrease in the number of mature neutrophils in these mice (Fig. 3D). This decrease in total neutrophil counts in U2AF1-S34F mice is likely due to a decrease in overall marrow cellularity in these mice, as the percentage of neutrophils in the marrow in the U2AF1-S34F mice was not significantly different than in U2AF1-wt mice (37.9 ± 2.1 WT vs 32.5 ± 2.1 S34F, mean ± SEM, *p* = 0.08). As reported previously [25], monocyte counts were significantly reduced in the U2AF1-S34F mice (75 ± 21 WT vs 13 ± 6 S34F, cells/ml blood × 10^3^, *p* = 0.03). Because blood and marrow neutrophils could both contribute to peritoneal infection-mediated neutrophil recruitment, it is possible that the diminished neutrophil counts in the marrow contribute, at least in part, to the neutrophil recruitment defect present in U2AF1-S34F mice.

**Fig. 4:**
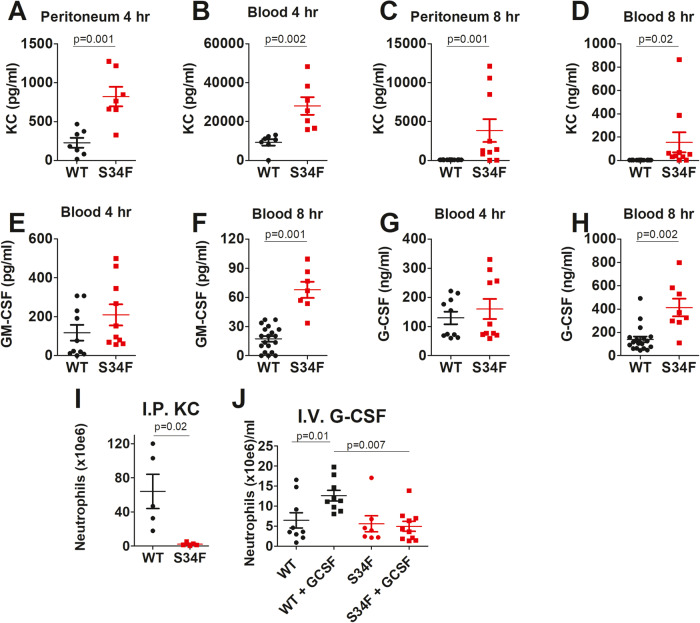
Neutrophils in U2AF1-S34F mice have an intrinsic cell migration defect. U2AF1-S34F, rtTA or U2AF1-wt, rtTA mice were injected daily for four days with 9-TB. **A**–**H** The mice were then infected with 10^8^ CFU *E. coli* via intraperitoneal injection. At the indicated times, the mice were humanely euthanized, and the indicated cytokines and chemokines were quantitated by ELISA in peritoneal lavage or blood. If no P value is listed in a particular panel, then the U2AF1-S34F and U2AF1-wt readings were not statistically different in that case. **I** The mice were injected via intraperitoneal injection with 500 ng KC. Four hours later, the number of infiltrating neutrophils was quantitated in peritoneal lavage. **J** The mice were injected with 3.12 µg G-CSF (or not as a control); 4 h later, the mice were humanely euthanized, and the number of neutrophils that emigrated into the blood were quantitated.

We next examined production of factors required for neutrophil migration, including KC, a chemokine involved in neutrophil recruitment, and GM-CSF and G-CSF, which are required for neutrophil maturation and emigration from the bone marrow [35, 36]. Production of all these factors was similar to or higher in mice expressing U2AF1-S34F compared to mice expressing U2AF1-wt following I.P. *E. coli* infection (Fig. 4A–H), indicating that extrinsic factors that drive neutrophil migration are present in U2AF1-S34F mice. This suggests that the neutrophil chemotaxis defect observed in U2AF1-S34F mice might be due to intrinsic defects in the neutrophils themselves.

To test if neutrophils in U2AF1-S34F mice were intrinsically deficient in migration, we injected the chemokine KC into the peritoneum of mice expressing U2AF1-S34F or U2AF1-wt. Even when the chemokine was introduced directly into these mice, U2AF1-S34F neutrophils failed to migrate into the peritoneum (Fig. 4I). In contrast, KC induced robust neutrophil recruitment in U2AF1-wt mice (Fig. 4I). Because both blood and bone marrow neutrophils could contribute to neutrophil recruitment, we also directly monitored neutrophil emigration from the bone marrow. When mice were injected intravenously (I.V.) with G-CSF, neutrophils emigrated from the bone marrow into the blood in mice expressing U2AF1-wt but not in mice expressing U2AF1-S34F (Fig. 4J). All these data suggest that neutrophils from U2AF1-S34F mice have an intrinsic migration defect.

**Fig. 5:**
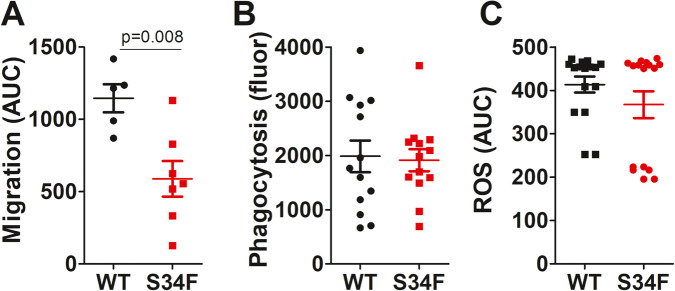
Neutrophils from U2AF1-S34F mice fail to migrate properly. U2AF1-S34F, rtTA or U2AF1-wt, rtTA mice were injected daily for four days with 9-TB. Bone marrow neutrophils were isolated and subjected to analysis ex vivo. **A** Neutrophils were fluorescently labeled, and neutrophil migration towards KC was quantified over the course of 90 min. Data are graphed in arbitrary cumulative fluorescence units (i.e., area under the curve, AUC). **B** Neutrophil uptake of fluorescently labeled *E. coli* particles over the course of 1 h was quantitated. Data in arbitrary fluorescence units. **C** Production of reaction oxygen species (ROS) was assessed by monitoring fluorescence of the ROS-reactive CM-H2DCFDA indicator for 2 h. Data in arbitrary cumulative fluorescence units (i.e., area under the curve, AUC). Data in graph panels that do not list a *p* value were not significantly different.

To confirm that the neutrophil migration defect in U2AF1-S34F mice is due to an intrinsic neutrophil defect, we purified neutrophils from mouse bone marrow and monitored chemotaxis towards KC in an ex vivo cell culture system. Isolated neutrophils from U2AF1-S34F mice migrated less efficiently towards KC than did neutrophils from U2AF1-wt mice (Fig. 5A). Even though neutrophils from U2AF1-S34F mice were defective in migration, they were not defective in all neutrophil functions. For example, neutrophils isolated from U2AF1-S34F mice phagocytosed *E. coli* particles and produced reactive oxygen species similarly to neutrophils from U2AF1-wt mice (Fig. 5B, C).

### Neutrophils from MDS patients carrying U2AF1 mutations exhibit a chemotaxis defect

**Fig. 6:**
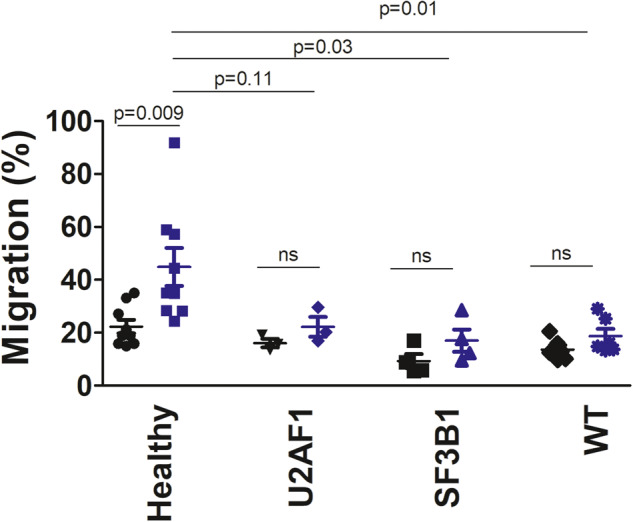
Neutrophils from MDS patients fail to migrate properly. Peripheral blood neutrophils were isolated from healthy blood donors and from patients with MDS. The MDS patient neutrophils were stratified by those harboring mutations in U2AF1, SF3B1, or those lacking any spliceosome mutation (WT). Neutrophils were fluorescently labeled, and neutrophil migration across a transwell in either the absence of stimulation (black dots) or the presence of 25 ng/ml IL-8 stimulation (blue dots) was quantified over the course of 90 min.

To determine if the neutrophil chemotaxis defect present in mice expressing U2AF1-S34F was also present in patients harboring this mutation, we isolated peripheral blood neutrophils from patients with MDS and from age and sex matched healthy volunteers (demographic information in Supplementary Tables 1 and 2, respectively). Neutrophil migration in a transwell cell culture system was monitored in either the absence of chemokines or in the presence of the chemotactic factor IL-8. IL-8 was able to significantly increase the migration of neutrophils from healthy donors (Fig. 6). In contrast, IL-8 did not significantly increase the migration of neutrophils isolated from MDS patients (Fig. 6). This migration defect was present in neutrophils from MDS patients that did not have a mutation in spliceosome genes and in neutrophils from patients that had mutations in either the U2AF1 or SF3B1 spliceosome genes (Fig. 6). Thus, MDS patient neutrophils exhibited a migration defect in all genotypes tested. These data suggest that multiple MDS-associated spliceosome mutations are capable of inducing neutrophil chemotaxis defects.

### Neutrophils from U2AF1-S34F mice have multiple defects in pathways controlling chemotaxis

To determine why neutrophils from U2AF1-S34F mice exhibit a cell migration defect, we first monitored production of the chemokine receptor CXCR2. Neutrophil migration is regulated by CXCR2, which responds to chemokines including IL-8 and GRO-α [37]. Surface levels of CXCR2 on neutrophils expressing U2AF1-S34F were largely unchanged as assessed by flow cytometry (Supplementary Fig. 3A). Likewise, ELISA analysis of CXCR2 levels in neutrophils from U2AF1-S34F mice identified a statistically significant decrease in CXCR2 levels in U2AF1-S34F mice; however, this decrease was fairly modest (Supplementary Fig. 3B), and its biological relevance is therefore unclear.

To determine why U2AF1-S34F neutrophils are defective in chemotaxis, we used RNA-seq to monitor the full complement of gene expression and mRNA splicing changes induced by U2AF1-S34F in neutrophils. Bone marrow neutrophils were isolated from mice expressing either U2AF1-S34F or U2AF1-wt, either in mice not infected or in mice infected with *E. coli* H9049 for 4 h. RNA was prepared from isolated neutrophils, and RNA-seq was performed. Principal component demonstrated that both mouse genotype and the presence or absence of infection altered gene expression in these neutrophils (Supplementary Fig. 4).

**Fig. 7:**
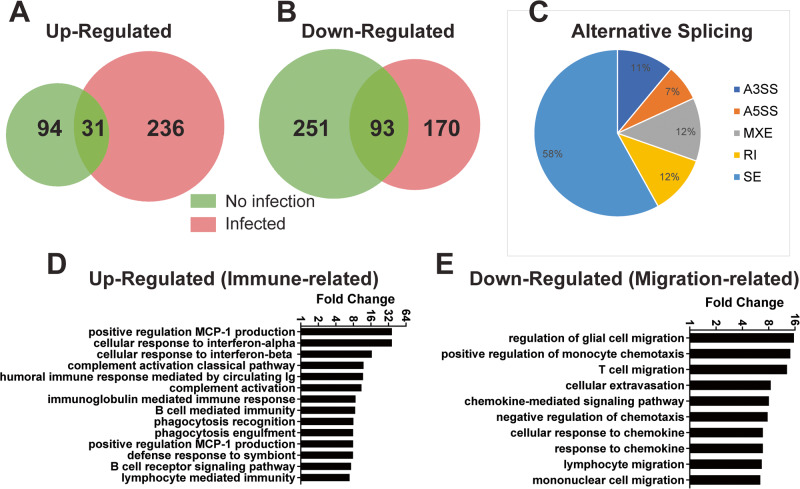
U2AF1-S34F induces changes in gene expression and alternative mRNA splicing in mouse neutrophils. Neutrophils were collected from mice expressing either U2AF1-S34F or control mice expressing U2AF1-wt, either in the absence of infection or in mice infected I.P. with *E. coli* strain H9049 for 4 h. RNA was isolated and gene expression and mRNA splicing were monitored using RNA-seq. Depicted are the number of genes that were up-regulated (**A**) or down-regulated (**B**) in neutrophils from U2AF1-S34F mice compared to neutrophils from U2AF1-wt mice in the absence (green) or presence (pink) of *E. coli* infection. **C** Alternative mRNA splicing events were identified using rMATs. The pie chart indicates the frequency of different alternative splicing events identified comparing neutrophils from U2AF1-S34F to U2AF1-wt mice (*N* = 8515 alternative splicing events in 3117 different genes). A3SS = alternative 3' slice site used. A5SS = alternative 5' splice site used. MXE = mutually exclusive exon usage. RI = retained intron. SE=skipped exon. **D** GO Pathway analysis was performed to identify pathways that were up-regulated in neutrophils from U2AF1-S34F mice compared to U2AF1-wt mice in the absence of infection. Fold change indicates the fold enrichment of that pathway relative to control. Pathways that affected the immune system were manually extracted in (**D**); the complete set of pathways that were up-regulated are listed in Supplementary Table 6. **E** GO Pathway analysis was performed to identify pathways that were down-regulated in neutrophils from U2AF1-S34F mice compared to U2AF1-wt mice in the absence of infection. Fold change indicates the fold enrichment of that pathway relative to control. Pathways that affected cell migration were manually extracted in (**E**); the complete set of pathways that were down-regulated are listed in Supplementary Table 8.

The presence of the U2AF1-S34F mutation (comparing neutrophils from U2AF1-S34F mice to neutrophils from U2AF1-wt mice) induced substantial changes in gene expression (Fig. 7A, B and Supplementary Table 3) and pre-mRNA splicing (Fig. 7C, Supplementary Table 4) in either the absence or presence of infection. These mRNA splicing changes included all classes of alternative splicing events, although approximately half of them were exon skipping events (Fig. 7C). We used Gene ontology (GO) analysis as a non-biased approach to identify pathways affected by U2AF1-S34F in neutrophils, comparing U2AF1-S34F to U2AF1-wt. Interestingly, GO-pathway analysis of mRNA splicing changes induced by U2AF1-S34F mice indicated that many RNA processing pathways were affected by the U2AF1 mutation (Supplementary Table 5). This observation is consistent with the observed effects of MDS-associated spliceosome mutations on alternative splicing in other cell types [32, 38, 39].

We also monitored the effects of U2AF1-S34F (compared to U2AF1-wt) on gene expression in neutrophils. In the absence or presence of infection, U2AF1-S34F increased the expression of 125 or 267 genes, respectively (Fig. 7A). More than half of the biological pathways upregulated in U2AF1-S34F neutrophils were immune-related pathways (Fig. 7D, Supplementary Tables 6 and 7). Among the GO categories over-represented in genes upregulated by U2AF1-S34F were “cellular response to interferon-alpha” and “cellular response to interferon-beta.” These data suggest that interferon signaling may be altered in neutrophils from U2AF1-S34F mice.

Pathway analysis of genes that were down-regulated by U2AF1-S34F in the absence or presence of infection also identified changes in immune-relevant pathways (Fig. 7B, Supplementary Tables 8 and 9). Notably, pathways relevant to cell migration and chemotaxis were down-regulated in neutrophils from U2AF1-S34F mice (Fig. 7E and Supplementary Tables 8 and 9), consistent with the chemotaxis defect observed in these mice. These migration-relevant pathways included but were not limited to “positive regulation of monocyte chemotaxis,” “lymphocyte migration,” and “mononuclear cell migration.” Other relevant pathways that were downregulated include “chemokine-mediated signaling pathway” and “cellular response to chemokine.” Finally, “cellular extravasation,” which could also affect neutrophil migration to the infection site, was altered [40–42]. Altogether, decreased expression in 18 different genes known to affect cell migration and chemotaxis were identified in this pathway analysis (Supplementary Fig. 5, Supplementary Table 10). Decreased expression of these 18 genes drove the pathway-level changes in cell migration observed (Fig. 7E). This suggests that decreased expression of these 18 genes could contribute to the neutrophil chemotaxis defect observed in U2AF1-S34F mice.

To confirm that gene expression in cell migration pathways was decreased in neutrophils from U2AF1-S34F mice, we also used a second pathway analysis platform, Ingenuity Pathway Analysis (IPA). IPA analysis of gene expression changes induced by U2AF1-S34F confirmed that cell migration pathways were downregulated in U2AF1-S34F neutrophils. Again, multiple gene expression changes contributed to the predicted decrease in expression of genes in these migration pathways (Supplementary Table 11). This analysis also suggested that downregulated chemotaxis-relevant gene expression could contribute to the cell migration defect in U2AF1-S34F neutrophils.

**Fig. 8:**
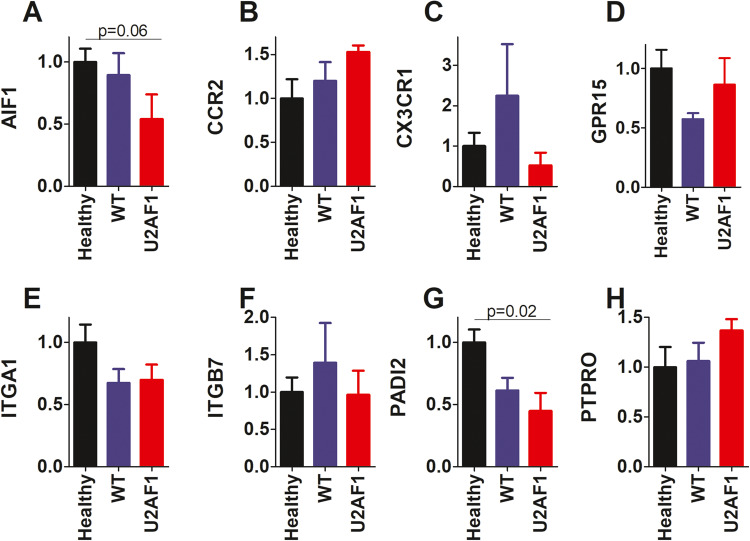
Expression of genes that regulate cell migration in neutrophils from MDS patients. Neutrophils were isolated from the peripheral blood of healthy blood donors (healthy, black bars) or MDS patients without any spliceosome mutations (WT, blue bars) or MDS patients with a U2AF1 mutation (U2AF1, red bars). Gene expression of the indicated genes was monitored by qPCR, with gene expression normalized so that expression in the healthy neutrophils was set to 1. *P* values that were statistically significant are indicated; lack of a *P* value indicates that comparison was not significantly different compared to control. *N* = (Healthy = 11, WT MDS = 5, U2AF1 MDS = 3).

To determine if any of these genes might also be contributing to the chemotaxis defect present in neutrophils from patients with MDS, we used qPCR to examine expression of the human homologs of these genes. 12 of the 13 genes with the largest decrease in expression in U2AF1-S34F mouse neutrophils had human orthologs (Supplementary Table 10). We used qPCR to examine expression of these 12 genes in human neutrophils; expression of 8 of these 12 genes was above background levels (Fig. 8). Compared to age and sex matched neutrophils from healthy donors, neutrophils from patients with U2AF1 mutations exhibited decreased expression of several of these genes, although only two (AIF1 and PADI2) approached or reached statistical significance, probably because of the small size of this human patient cohort (Fig. 8). It is possible that U2AF1-mutation induced changes in expression of these genes contribute to the chemotaxis defect present in neutrophils from MDS patients.

## Discussion

MDS patients are known to have heightened susceptibility to infection, and infection is among the most common causes of death in these patients. Some MDS patients have significant neutropenia, which contributes to this host defense defect. Even in patients that have normal neutrophil counts, significant deficits in neutrophil function have been reported. For example, neutrophils from some patients are reported to exhibit a weakened ability to phagocytose bacteria [11, 13, 16], decreased production of important antimicrobial compounds such as elastase, myeloperoxidase, and superoxide [11–13, 43–46], and decreased production of neutrophil extracellular traps [47]. These and other defects likely contribute to the decreased bactericidal capacity of neutrophils from MDS patients [13, 16, 48]. Moreover, neutrophils from MDS patients also exhibit defects in migration towards chemotactic signals [11, 14–16, 44, 49–51]. While there are reports that some of these functional defects correlate with disease status, how mutation status in these patients impacts neutrophil function, and relatedly, what are the molecular drivers of this neutrophil dysfunction has not been addressed previously.

In the current study, we found that mice engineered to express U2AF1-S34F but not wild type U2AF1 exhibit a profound immunodeficiency, likely due to neutrophil chemotaxis defects induced by this MDS-associated spliceosome mutation. U2AF1-S34F mice were unable to control a peritoneal *E. coli* infection, with recoverable CFU counts comparable to what was instilled into these mice. In contrast, wild type mice were able to control this infection very rapidly. Despite this profound immunodeficiency, U2AF1-S34F mice were able to recognize the presence of the infection, as the U2AF1-S34F mice produced pro-inflammatory cytokines as well as factors that drive neutrophil migration and bone marrow emigration. Importantly, in U2AF1-S34F mice, neutrophils failed to migrate to the infection site, suggesting that neutrophils from U2AF1-S34F mice were chemotaxis deficient. The decreased number of bone marrow neutrophils in U2AF1-S34F mice may contribute, at least in part, to this defect. However, neutrophils were present at levels similar to wild type in blood in the U2AF1-S34F mice, and these cells failed to migrate to the infection site, suggesting an intrinsic neutrophil migration defect. This was confirmed by monitoring neutrophil chemotaxis towards KC both in vivo and ex vivo. The neutrophils that were present in bone marrow in U2AF1-S34F mice failed to emigrate into the blood after I.V. G-CSF treatment, also consistent with an intrinsic neutrophil functional defect in these mice. Finally, studies using neutrophils from MDS patients and healthy control subjects suggested that neutrophils from MDS patients, regardless of genotype and including U2AF1-S34F mutations, were also intrinsically chemotaxis defective. These data imply that multiple MDS-associated mutations including mutations in spliceosome genes can induce neutrophil chemotaxis defects. Importantly, our mouse studies demonstrate that the MDS-associated U2AF1-S34F mutation is sufficient to induce this defect.

How does the MDS-associated U2AF1-S34F mutation impact neutrophil chemotaxis? Our studies indicated that there is at most a fairly limited decrease in levels of the chemokine receptor CXCR2 in U2AF1-S34F mice, suggesting that U2AF1-S34F likely impacts other downstream cell migration factors. This observation is in agreement with a study of the migration of human neutrophils from MDS patients, in which it was observed that the neutrophil migration rate in these patients did not correlate with chemokine receptor levels [49]. This result also implicates downstream mediators as responsible for the neutrophil migration deficit present in MDS patients.

We used RNA-seq to identify changes in gene expression and pre-mRNA splicing in neutrophils that could drive this U2AF1-S34F-induced chemotaxis defect, comparing neutrophils isolated from mice expressing U2AF1-S34F to neutrophils from mice expressing U2AF1-wt. The U2AF1 mutation induced changes in splicing of genes in numerous pathways involved in RNA processing. Similar pathways are reported to be altered by MDS-associated spliceosome mutations in other cell types, suggesting that this may be one common effect of this class of mutation [32, 38, 39]. At the gene expression level, many immune signaling pathways exhibited altered gene expression induced by U2AF1 mutation, in either the absence or presence of infection. Strikingly, multiple signaling pathways involving cell migration were downregulated in neutrophils from U2AF1-S34F mice. These pathway-level changes were driven by decreased expression of at least 18 genes. We monitored expression of the human orthologues of a handful of these genes to determine if similar changes in gene expression were induced by U2AF1 mutation in human neutrophils. Expression of two of these genes, AIF1 and PADI2, were similarly downregulated in neutrophils from MDS patients harboring U2AF1 mutations. One limitation of these studies is the small size of the human patient cohort, due to the logistics of collecting samples prospectively to isolate neutrophils. Compounding this issue is the fact that MDS is a heterogeneous disease. Thus, it is possible that some of the other candidate genes that regulate neutrophil migration might also be significantly downregulated if examined in a larger cohort.

Both AIF1 and PADI2 have been implicated in the regulation of immunity and cell migration and could contribute to the neutrophil migration defect in U2AF1-S34F mice and MDS patients. AIF1 is a calcium binding protein that affects inflammation [52]. AIF1 facilitates cell migration in multiple cell types [53–57], possibly by cross-linking actin [58, 59]. PADI2 is a peptidylarginine deiminase, an enzyme that converts arginine into citrulline. This peptide modification can have significant effects on protein function including regulators of the immune response [60, 61]. PADI2 regulates T cell migration by citrullinating various chemokines [62] and is also reported to affect various innate immune signaling pathways in myeloid cells [63–66].

While our studies demonstrate that U2AF1-S34F has profound consequences for neutrophil function, other immune cell types may also be affected by this mutation. MDS is known to affect multiple immune cell types [67], although these defects have not been characterized as thoroughly as MDS-associated neutrophil defects. For example, macrophages [68] from MDS patients are reported to have some deficiencies. It is unknown if MDS-associated U2AF1 mutations affects other myeloid cells.

It is also unknown if the immunodeficiency induced by MDS-associated U2AF1 mutations is unique to U2AF1 or if other spliceosome mutations induced similar defects. Prior studies demonstrated that there were overlaps in gene expression changes induced by these different spliceosome mutations, particularly at the pathway level [32, 38, 39]. Therefore, it is possible that similar host defense defects could be induced by these mutations, although this remains to be tested.

There has been a temporal disconnect in the studies of myeloid cell defects in MDS patients, as many of the studies focused on the function of these myeloid cells predate the identification of the panoply of mutations that drive this cancer, and thus, also predate the development of transgenic mouse models expressing these MDS-associated mutations. Thus, to our knowledge, this is the first study that directly examines how an MDS-associated mutation that drives MDS impacts myeloid cell function and host defense. In particular, our study demonstrates that U2AF1 mutations found in MDS patients are sufficient to induce functional neutrophil defects in mice. This effect likely contributes to the increased infection risk and infection-associated morbidity in patients with MDS.

### Supplementary information

Supplementary Information

Supplementary Table 3

Supplementary Table 4

## Data Availability

The RNAseq data generated in this study have been deposited in the Gene Expression Omnibus Database under GEO Accession number GSE209799. All other data are present in the manuscript figures, tables, and supplementary files.

## Code availability

Our studies use publicly or commercially available software packages for all analyses as outlined in the Supplementary Methods.
