# Supplementary material for: Myelodysplastic neoplasm-associated U2AF1 mutations induce host defense defects by compromising neutrophil chemotaxis
Source: Leukemia. 2023 Aug 17;37(10):2115–24. doi: 10.1038/s41375-023-02007-7 (PMC10539173; doi:10.1038/s41375-023-02007-7)
Supplement: Supplementary file 1 — Supplementary Information [file 41375_2023_2007_MOESM1_ESM.pdf]

GURULE ET AL. SUPPLEMENTARY INFORMATION

Supplementary Methods

Supplementary References

Supplementary Figure Legends

Supplementary Figures 1-6

Supplementary Tables 1, 2, 5-11

Supplementary Tables 3 and 4 are provided as separate Excel files

Supplementary Methods

Assessment of bacterial killing by 9-TB

E. coli strain H9049 [1-3] was grown in LB medium and was then washed and diluted in 0.9% NaCl. Tubes containing 0.1 ml LB were prepared with 10-fold serial dilutions of 9-TB (9-tert-Butyl Doxycyline from Echelon Biosciences, catalog B-0801) or tobramycin (a control antibiotic, Fresenius Kabi catalog #300602) at the indicated concentrations. Tubes were inoculated with 5 x 10^6 CFU *E. coli* and incubated for 24 hours at 37°C. Cultures were then subjected to serial dilution and plated on LB agar; CFU counting subsequently was used to determine the effect of the two agents on bacterial growth.

Mouse infection studies

For mouse infection studies, mice were injected via intraperitoneal injection with 10^8 CFU of *E. coli* strain H9049 [1-3]. To analyze host defense in these mice, mice were humanely euthanized, and the effects of U2AF1 transgene expression on host defense were analyzed. Peritoneal cells were harvested by peritoneal lavage with sterile PBS supplemented with 1 mM EDTA and 10 mM HEPES (pH 7.2) at the indicated times. Cell counts were determined by counting using a hemocytometer. Cell differentials were determined by staining cytospin slides. Mouse blood was collected by cardiac puncture in 0.5 M EDTA. Spleen and liver also were isolated in some studies.

Viable bacteria were quantified by counting colony forming units (CFUs). Peritoneal lavage cell suspension was diluted in 500 µl of 0.2% Triton X-100. Serial dilutions (10^{-2} to 10^{-5}) were made using 450 µl 1X PBS and 50 µl cell suspension in Triton X-100. On LB plates, 20 µl of diluted culture was plated in each quadrant and incubated over night at 37ºC. Colonies were counted the next day, and data are presented as CFU/ml. Organs were homogenized using a tissue homogenizer prior to serial dilution and plating.

In other studies, mouse morbidity was assessed over the course of several days with the infection starting at day 0.

For mouse studies, 8-12 week old male and female mice were randomly selected for each experiment based on availability. U2AF1-S34F and U2AF1-wt mice were age and sex-matched in each study; no mice were excluded from the study. No blinding was performed. The number of mice used is indicated in each figure and/or figure legend.

Bone marrow transplants

Bone marrow transplants were performed as previously described [5, 6]. Radiation was provided to wild type recipient mice as 900 cGy of total body radiation from a ^{137}Cs source. Adoptive transfer of donor bone marrow took place six hours after irradiation. Bone marrow cells were obtained from the hind limbs of donor mice by flushing the cells from the marrow using sterile media. Cell suspensions were centrifuged for 10 minutes at 200 g and decanted. Pellets were resuspended in sterile HBSS, filtered through a 40-μm mesh, and centrifuged at 200 g. Following a third wash step, the cells were resuspended in HBSS at a concentration of $25x10^7$ cells/ml. Recipient mice were injected with $5x10^6$ bone marrow cells in 200 μl HBSS via the lateral tail vein. Engrafted mice were subsequently analyzed six weeks after bone marrow transplantation.

Quantitation of neutrophil recruitment to the peritoneum following KC administration

8-12 week old mice were injected via I.P. injection with 500 ng recombinant mouse KC (R&D Biosystems catalog # 453-KC). 4.5 hours later, peritoneal cells were harvested by peritoneal lavage with sterile PBS supplemented with 1 mM EDTA and 10 mM HEPES (pH 7.2). Cells were counted and cytospin slides were stained as described above to quantitate the number of recruited cells.

Quantitation of neutrophil mobilization to the peripheral blood following G-CSF administration

8-12 week old mice were administered 3.12 μg mouse recombinant G-CSF (R&D Biosystems catalog #414-CS) or PBS control through tail vein injections. 4 hours later, blood was collected from the heart with heparinized syringes and transferred immediately to 10 ml red blood cell lysis buffer for 10 min at 4º C. Cells were collected by centrifugation, cell counts were quantitated, and the cells were then processed for flow cytometry analysis.

Isolation of mouse neutrophils

Neutrophils used for the analysis of chemotaxis were isolated by a three-density gradient as described previously [7]. Briefly, mice were euthanized, and bone marrow was harvested by flushing the femurs and tibias with Hank's balanced salt solution (HBSS). The marrow was washed and placed on top of 72, 64, and 52% layers of Percoll in HBSS, and the gradient was centrifuged at 1,060 g for 30 min. Neutrophils formed a band at the interface of the 64 and 72% Percoll layers, and were morphologically >95% pure. This band was carefully aspirated, washed, and resuspended in Krebs-Ringer phosphate solution containing 0.2 % dextrose (KRPD).

Neutrophils used for other functional studies were isolated using the mouse neutrophil isolation kit (Miltenyi Biotec catalog # 130-097-658). Bone marrow was collected from the tibias and femurs of mice. Red blood cells were lysed, and magnetic labeling of non-target cells was performed according to the manufacturer's instructions. Magnetic separation was performed using a MACS LS Column and a QuadroMacs separation magnet (Miltenyi Biotec). The flow through, which contained the enriched neutrophils, was collected. Neutrophil purity was confirmed by staining of cytospin slides.

Quantitation of neutrophil chemotaxis

To monitor mouse neutrophil chemotaxis, purified mouse bone marrow neutrophils were labeled with calcein-AM as previously described [8]. Briefly, 2 x 10^6 bone marrow neutrophils were suspended in 0.5 ml KRPD supplemented with 2% human heat-inactivated platelet poor plasma (HIPPP) and labelled in 5 µM calcein-AM for 15 min at 37°C. The neutrophils were then washed once and resuspended in 0.5 ml KRPD/HIPPP. Labeled cells were placed in the upper chamber of a FluoroBlok 96-well Multiwell Insert System (Corning), and KRPD/HIPPP was placed in the lower chamber. Cell migration was initiated with the addition of 500 ng/ml KC (R&D Biosystems) to the lower chamber; as a control, non-directional migration was monitored in the absence of chemokine addition to the lower chamber. Fluorescence measurements were made at an excitation of 488 nm and emission of 528 nm using a BioTek FLx800 plate reader every 2 min over a 90 min period. Cumulative migration was quantitated in area under the curve (AUC) of arbitrary fluorescence units.

To monitor human neutrophil chemotaxis, a similar procedure was used. Purified neutrophils were suspended in 0.5 ml KH buffer and then stained in 5 µM Calcein-AM by incubating at 37ºC for 10 min. Cells were washed once with Krebs-Ringer phosphate-dextrose (KRPD) buffer and were subsequently plated in the upper chamber in trans-well plates at a density of 100,000 cells/well in 100 µl total volume. 25 nM IL-8 or KH buffer (control) was included in the lower chamber (total volume 100 µl). Absorbance at 485/520 (Ex/Em) from the bottom of the plate was measured every 2 min for 90 min using a plate reader. To calculate 100% migration efficiency, the labeled neutrophils were plated into the lower chamber of a well and fluorescence was monitored immediately.

Quantitation of neutrophil phagocytosis

Freshly isolated mouse bone marrow-derived neutrophils were used. 100,000 neutrophils/well were seeded in a 96 wells plates with opaque sides and incubated at 37ºC for 1

hour in 1X PBS supplemented with 0.5% bovine serum albumin (BSA) and 2 mM EDTA. Phagocytic capacity of the neutrophils was then assessed using the Vybrant Phagocytosis Assay kit (Thermofisher Scientific catalog #V6694) according to the manufacturer's instructions. Uptake of FITC-labeled *E. coli* particles was quantitated using a plate reader and was reported in arbitrary fluorescence units.

Quantitation of neutrophil ROS production

Freshly isolated mouse bone marrow-derived neutrophils were used. Reactive oxygen species (ROS) were detected using the cell permeant CM-H2DCFDA indicator (ThermoFisher Scientific catalog #C6827). Purified neutrophils were suspended at $4x10^6$ cells per ml in RPMI supplemented with 10 mM HEPES pH 7.5, 1% HIPP (RHH). Cells were stained with 10 µm CM-H2DCFDA by incubating at room temperature for 20 minutes, washed once with RHH, and re-suspended in 1 ml RHH. Labeled neutrophils were plated at 200,000 cells per well in a 96-well black-walled plate and centrifuged at 110 x g for 2 min at room temperature. Absorbance at 458/528 nM was measured at 37ºC every 10 min for 2 hours. Data is depicted as AUC for the cumulative time of stimulation.

Flow cytometry analysis

Blood and peritoneal lavage were analyzed as follows. Cells were re-suspended in HBSS supplemented with 30% BSA and 0.5 M EDTA (complete buffer). Cell suspensions were incubated with unlabled CD16/CD32 (1:100 for 5 min) to block non- specific FcɣR-mediated binding. Cells were stained with surface antibodies at 1:200 for 45 min and then washed once with complete buffer. All incubations were performed in the dark at 4ºC. The surface antibody panel used included CD45-FITC (BD #553080), CD11b-BUV395 (BD #563553), Ly6C-APC (Biolegend #128016), Ly6G-APC-Cy7 (Biolegend #127624), CD115-PE (Invitrogen #12-1152-

82), CD3-PB (Biolegend # 100214), and CD19-PE-Cy7 (Invitrogen #25-0193-82). Stained cells were fixed with 4% PFA in PBS for 15 min at 4° C in the dark, washed once in complete buffer, and then re-suspended for FACS analysis using a BD Biosciences LSR II Flow Cytometer.

Bone marrow population were analyzed as follows: marrow from both femurs and tibiae was flushed using staining media (SM; Hanks' Buffered Saline Solution + 2% FBS) and a 3 ml syringe fitted with a 21-gauge needle. After flushing, the cells were resuspended in 1 ml of 1X ACK (Ammonium-Chloride Potassium Lysing Buffer) to remove erythrocytes, for five minutes on ice. The cells were subsequently washed with SM and filtered through a 70 µm mesh filter. Each sample was then resuspended in SM at a 1:50 dilution, and the cells were counted on a Vicell automated counter (Beckman Coulter). For quantification of mature bone marrow cells, purified rat IgG (Sigma-Aldrich #18015-100MG) was added as a blocking agent in addition to the antibodies: Siglec-1-FITC (Biolegend #142405), CD115-A488 (Biolegend #135511), CD101-PE (ThermoFisher #12-1011-80), Ly6G-PE/Cy7 (Biolegend #127617), CXCR2-APC (Biolegend #149312), B220-A700 (Biolegend #103232), Ly6C-APC/Cy7 (Biolegend #128025), Gr1-PB (Biolegend #108430), and CD11b (Mac 1)-BV605 (Biolegend #101237) with Brilliant Buffer (BD Biosciences #563794). Following the addition of the stain, the cells were incubated in the dark for 30 minutes on ice. Following surface staining, the samples were washed with SM and resuspended in SM containing 1 µg/ml propidium iodide (SM PI; staining media plus propidium iodide) to distinguish live and dead cells. Immediately following staining with SM PI, the cells were analyzed using a FACSCelesta flow cytometer (BD). Flow data was analyzed using FlowJo, Microsoft Excel, and Graphpad Prism.

Sample data from U2AF1-wt and U2AF1-S34F mice are presented in Supplementary Fig. 6. Total neutrophils are Ly6G$^+$; mature neutrophils are additionally CD101$^+$ [9].

Isolation and sequencing of RNA from mouse neutrophils

Total RNA was isolated using standard kits from Qiagen (Valencia, CA, USA). The isolated total RNA was processed for next-generation sequencing (NGS) library construction as developed in the National Jewish Health Genomics Facility for analysis with an Illumina NovaSeq 6000 (San Diego, CA, USA). A Takara Bio (Mountain View, CA, USA) Smart-Seq v4 Ultra Low Input RNA kit for whole transcriptome cDNA generation was used in conjunction with an Illumina (San Diego, CA, USA) Nextera XT DNA Library Prep kit to primarily target all polyA RNA. Briefly, library construction started from isolation of total RNA species, followed by proprietary SMARTer first strand cDNA synthesis, full length doubled stranded cDNA synthesis via LD-PCR, cDNA amplification, fragmentation and adapter ligation, and library amplification. Once validated, the libraries were sequenced as barcoded-pooled samples and run on the NovaSeq 6000 on a S4 flowcell with 2x150bp sequencing chemistry.

RNA-seq data analysis

Fastqc (0.11.5) was used to assess the quality of the fastq files. They were subsequently trimmed with skewer (0.2.2) of adapter sequence and cut to 140 bp [10]. Rmats requires reads of the same length so the fastq files were trimmed again to 140 bp as most reads reached this length and shorter reads were discarded. The two-pass STAR (2.7.5a) mapping method was used [11]. Initially all fastq files were mapped together to Mus_musculus.GRCm39.dna.primary_assembly.fa genome. The resulting junction file, SJ.out.tab, was filtered in R (4.1.0 "Bunny-Wunnies Freak Out") to remove MT chromosome splice junctions and unannotated splice junctions supported with less than three reads. These splice annotations were used to generate a new STAR genome and all paired fastq files were individually mapped. Coordinate sorted bam files were generated for later Rmats use using samtools (1.5). STAR generated individual count tables were combined and differential gene expression was performed using EdgeR (3.32.1). Stringtie (2.1.4) was used to generate individual gtf files from the mapped bam file and then merged into one gtf file [12]. Rmats (4.1.0) was then used to discover differential alternative splicing [13].

For Gene Ontology analysis, genes were sorted based on False Discovery Rate (FDR) of 0.05 or greater. Gene expression values were then filtered for logFC greater than zero for upregulated genes and logFC less than zero for downregulated genes. The resulting gene lists were submitted to Panther version 17.0 Gene Ontology Enrichment Analysis (http://www.pantherdb.org) [14, 15] using the overrepresentation test, Fisher test type, with FDR correction. The top 25 significant pathways are reported for each comparison.

Pathway analysis was also performed using the "diseases and biological functions" analysis in Ingenuity Pathway Analysis (IPA, Qiagen) [16] using an FDR cutoff of 0.05 and a fold-change cutoff of 1.7.

Isolation of peripheral blood neutrophils from patients with MDS

The collection and analysis of human peripheral blood samples was approved by the National Jewish Health Institutional Review Board (IRB) and the Colorado Multiple Institutional Review Board. All subjects gave informed written consent prior to participation.

Peripheral blood from untreated patients with MDS was collected as part of an IRB-approved tissue acquisition protocol at the University of Colorado. Patient clinical parameters were collected through retrospective chart review as described [17]. Patient sample genotyping for MDS-relevant genes was performed using a targeted next-generation sequencing panel used for patient diagnostics at the University of Colorado Cancer Center [17].

Peripheral blood from healthy control subjects was provided by Vitalant. Healthy control subjects were > 60 years of age. These healthy volunteers answered a questionnaire to verify that they were not experiencing symptoms of acute infection, did not have underlying chronic conditions, and were not taking corticosteroids. The age of the patients with MDS was 71.3±1.6; the age of the healthy donors was 68.0±2.1 (mean + SEM).

Peripheral blood neutrophils were isolated using the MACSxpress whole blood neutrophil isolation kit (Miltenyi Biotec catalog #130104434). MACS Express Beads were re-suspended according to the manufacturer's instructions and mixed with 8 ml of fresh whole blood. Non-target cells were labeled by incubating for 5 min at room temperature with agitation. Neutrophils were isolated from non-target cells using a MACS Express Separator magnet for 15 minutes. Supernatant, which contains the enriched neutrophils, was recovered. Cells were pelleted and washed with 1X PBS. Hypotonic lysis of red blood cells was performed by re-suspending the cell pellet in 0.9 ml of water for 15 sec followed by adding 0.1 ml of 10x PBS to quench the lysis. Cells were centrifuged for 1 min at 4000 x g, and the pellet was re-suspended in 10 ml KH [KRPD/1% HIPPP (heat-inactivated platelet-poor plasma)]. Neutrophil purity was confirmed by staining cytospin slides.

The absence of prior studies on patient genotyping and neutrophil function precluded an accurate power estimation; therefore, all available blood samples from consenting, untreated MDS patients were analyzed for neutrophil function. Most but not all of the MDS patient sample analysis was blinded, as provided blood samples were analyzed prior to reporting of patient genotyping.

ELISA and qPCR analyses

ELISAs were performed on isolated mouse peritoneal lavage and mouse blood using kits provided by R&D Biosystems according to the manufacturer's instructions. These included: IL-6 (catalog # DY406), TNFα (DY410), KC (DY453), GM-CSF (DY415), and G-CSF (DY414).

To quantitate CXCR2 levels in neutrophils, an ELISA kit from Novus Biologicals (catalog # NBP3-06771) was used. Cell lysis of neutrophils was performed with three consecutive liquid nitrogen freeze thaw cycles. Lysates were assayed according to the manufacturer's instructions. Each lysate was generated by pooling neutrophils from three mice. Absorbance was measured

at 450 nm. The concentration of CXCR2 protein was normalized to the total cellular protein per sample, and the data are presented as pg/μg.

RNA for qPCR was purified using RNAeasy mini kits (Qiagen). qPCR was performed using the Quantitect SYBR-Green RT-PCR kit (Qiagen) on a QuantStudio 7 Flex (Applied Biosystems). Gene expression levels were normalized relative to βactin using the ddCt method. Oligonucleotide sequences for the qPCR are listed in Supplementary Table S10. Human βactin primer sequences were those described in [18].

Statistics

GraphPad Prism was used to generate graphs and perform statistical analyses. Unless otherwise noted, data are graphed as the mean ± standard error of the mean (SEM) of at least three independent biological replicates. A one- or two-way ANOVA was used to compare differences in more than two groups. An unpaired, two tailed t-test was used to compare differences between two groups in data with a normal distribution. Normality was assessed using the Shapiro-Wilk test. Survival data was analyzed using the Log-rank (Mantel-Cox test). Statistical significance was consider $p<0.05$. Venn diagrams were generated using BioVenn [19].

Supplementary References

1. Fischbach MA, Lin H, Zhou L, Yu Y, Abergel RJ, Liu DR, et al. The pathogen-associated iroA gene cluster mediates bacterial evasion of lipocalin 2. Proceedings of the National Academy of Sciences of the United States of America. 2006;103(44):16502-7.
2. Flo TH, Smith KD, Sato S, Rodriguez DJ, Holmes MA, Strong RK, et al. Lipocalin 2 mediates an innate immune response to bacterial infection by sequestrating iron. Nature. 2004;432(7019):917-21.
3. Litvak V, Ramsey SA, Rust AG, Zak DE, Kennedy KA, Lampano AE, et al. Function of C/EBPdelta in a regulatory circuit that discriminates between transient and persistent TLR4-induced signals. Nature immunology. 2009;10(4):437-43.
4. Shirai CL, Ley JN, White BS, Kim S, Tibbitts J, Shao J, et al. Mutant U2AF1 Expression Alters Hematopoiesis and Pre-mRNA Splicing In Vivo. Cancer cell. 2015;27(5):631-43.
5. Janssen WJ, Barthel L, Muldrow A, Oberley-Deegan RE, Kearns MT, Jakubzick C, et al. Fas determines differential fates of resident and recruited macrophages during resolution of acute lung injury. American journal of respiratory and critical care medicine. 2011;184(5):547-60.
6. Janssen WJ, Muldrow A, Kearns MT, Barthel L, Henson PM. Development and characterization of a lung-protective method of bone marrow transplantation in the mouse. Journal of immunological methods. 2010;357(1-2):1-9.
7. Nick JA, Young SK, Brown KK, Avdi NJ, Arndt PG, Suratt BT, et al. Role of p38 mitogen-activated protein kinase in a murine model of pulmonary inflammation. Journal of immunology. 2000;164(4):2151-9.
8. Nick JA, Coldren CD, Geraci MW, Poch KR, Fouty BW, O'Brien J, et al. Recombinant human activated protein C reduces human endotoxin-induced pulmonary inflammation via inhibition of neutrophil chemotaxis. Blood. 2004;104(13):3878-85.

9. Evrard M, Kwok IWH, Chong SZ, Teng KWW, Becht E, Chen J, et al. Developmental Analysis of Bone Marrow Neutrophils Reveals Populations Specialized in Expansion, Trafficking, and Effector Functions. Immunity. 2018;48(2):364-79 e8.
10. Jiang H, Lei R, Ding SW, Zhu S. Skewer: a fast and accurate adapter trimmer for next-generation sequencing paired-end reads. BMC bioinformatics. 2014;15:182.
11. Dobin A, Davis CA, Schlesinger F, Drenkow J, Zaleski C, Jha S, et al. STAR: ultrafast universal RNA-seq aligner. Bioinformatics. 2013;29(1):15-21.
12. Kovaka S, Zimin AV, Pertea GM, Razaghi R, Salzberg SL, Pertea M. Transcriptome assembly from long-read RNA-seq alignments with StringTie2. Genome biology. 2019;20(1):278.
13. Shen S, Park JW, Lu ZX, Lin L, Henry MD, Wu YN, et al. rMATS: robust and flexible detection of differential alternative splicing from replicate RNA-Seq data. Proceedings of the National Academy of Sciences of the United States of America. 2014;111(51):E5593-601.
14. Thomas PD, Ebert D, Muruganujan A, Mushayahama T, Albou LP, Mi H. PANTHER: Making genome-scale phylogenetics accessible to all. Protein Sci. 2022;31(1):8-22.
15. Mi H, Muruganujan A, Thomas PD. PANTHER in 2013: modeling the evolution of gene function, and other gene attributes, in the context of phylogenetic trees. Nucleic acids research. 2013;41(Database issue):D377-86.
16. Kramer A, Green J, Pollard J, Jr., Tugendreich S. Causal analysis approaches in Ingenuity Pathway Analysis. Bioinformatics. 2014;30(4):523-30.
17. Pollyea DA, Hedin BR, O'Connor BP, Alper S. Monocyte function in patients with myelodysplastic syndrome. Journal of leukocyte biology. 2018;104(3):641-7.
18. Pollyea D, Kim H, Stevens B, Lee F, Harris C, Hedin B, et al. MDS-associated SF3B1 Mutations Enhance Pro-Inflammatory Gene Expression in Patient Blast Cells. J Leuk Biol. 2021;110(1):197-205.

19. Hulsen T, de Vlieg J, Alkema W. BioVenn - a web application for the comparison and visualization of biological lists using area-proportional Venn diagrams. BMC genomics. 2008;9:488.

Supplementary Figure Legends

Supplementary Fig. 1. The doxycycline analog 9-TB does not exhibit significant antimicrobial activity. *E. coli* strain H9049 was incubated for 24 hours at 37°C in the presence of the indicated concentrations (in µg/ml) of the doxycycline analog 9-TB (black bars) or the control antibiotic tobramycin (red bars). Viable bacteria were quantitated by counting colony forming units (CFUs) after this incubation.

Supplementary Fig. 2. U2AF1-S34F mice produce pro-inflammatory cytokines in response to infection. U2AF1-S34F, rtTA mice and U2AF1-wt, rtTA mice were injected with 9-TB daily for four days, then were exposed to 10^8 CFU *E. coli* strain H9049 via intraperitoneal injection. Either four or eight hours after infection, as indicated, TNFα and IL-6 protein production was assessed by ELISA in peritoneal lavage and blood. * indicates that the WT and S34F readings were significantly different ($P<0.05$). Data in panels without an asterisk were not significantly different.

Supplementary Fig. 3. Quantitation of CXCR2 levels in neutrophils from U2AF1-S34F mice. CXCR2 levels were analyzed by flow cytometry in mature neutrophils in bone marrow as described in Fig. 3 (panel **A**) or by ELISA (panel **B**). CXCR2 gMFI flow data was normalized to 1 in U2AF1-wt mice.

Supplementary Fig. 4. Gene expression in neutrophils is affected by U2AF1 mutation status and infection status. Depicted is a principal components analysis of gene expression changes induced in neutrophils expressing either U2AF1-S34F or U2AF1-wt, either in the presence (Yes) or absence (No) of peritoneal *E. coli* infection.

Supplementary Fig. 5. Many genes that regulate cell migration are down-regulated in neutrophils from U2AF1-S34F mice. Neutrophils were isolated from mice expressing either U2AF1-S34F or U2AF1-wt, RNA was prepared, and RNA-seq was used to monitor gene expression. Gene expression of the indicated genes that regulate cell migration is depicted as counts per million (CPM). * indicates a statistically significant difference in gene expression. N=4.

Supplementary Fig. 6. Gating scheme for analysis of mouse bone marrow neutrophils. Depicted are representative flow cytometry plots depicting total neutrophils (left column) and mature neutrophils (right column) in U2AF1-wt (top) or U2AF1-S34F (bottom) mice.

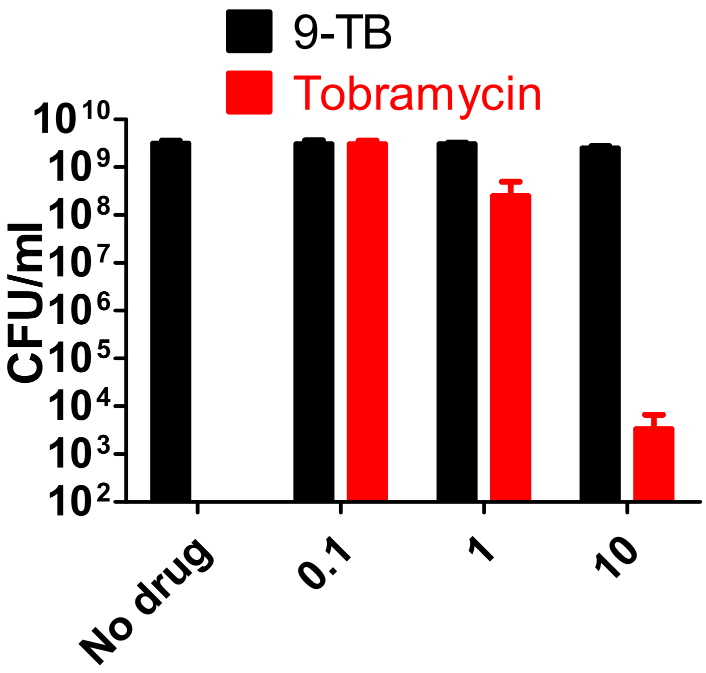

Supplementary Figure 1

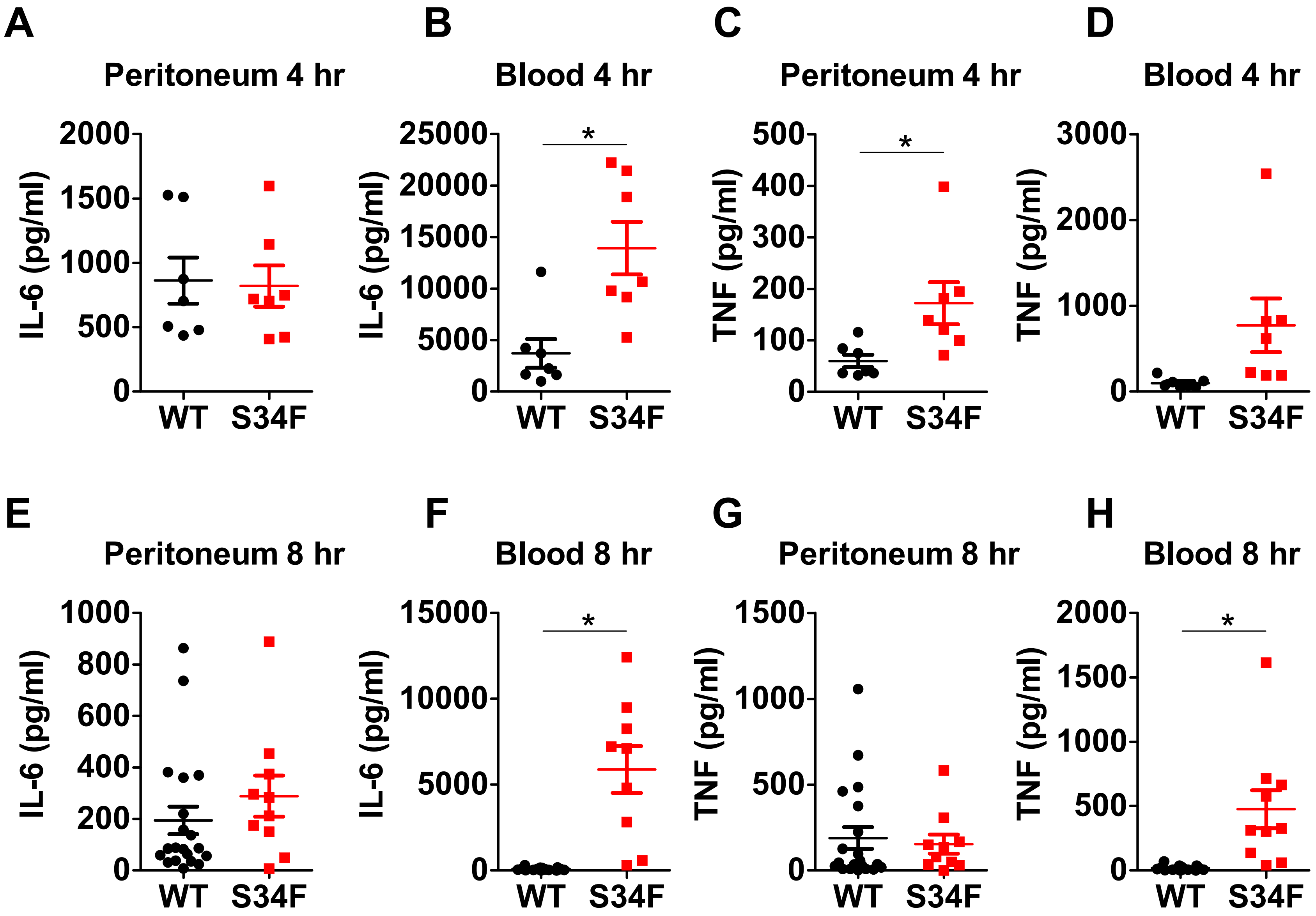

Supplementary Figure 2

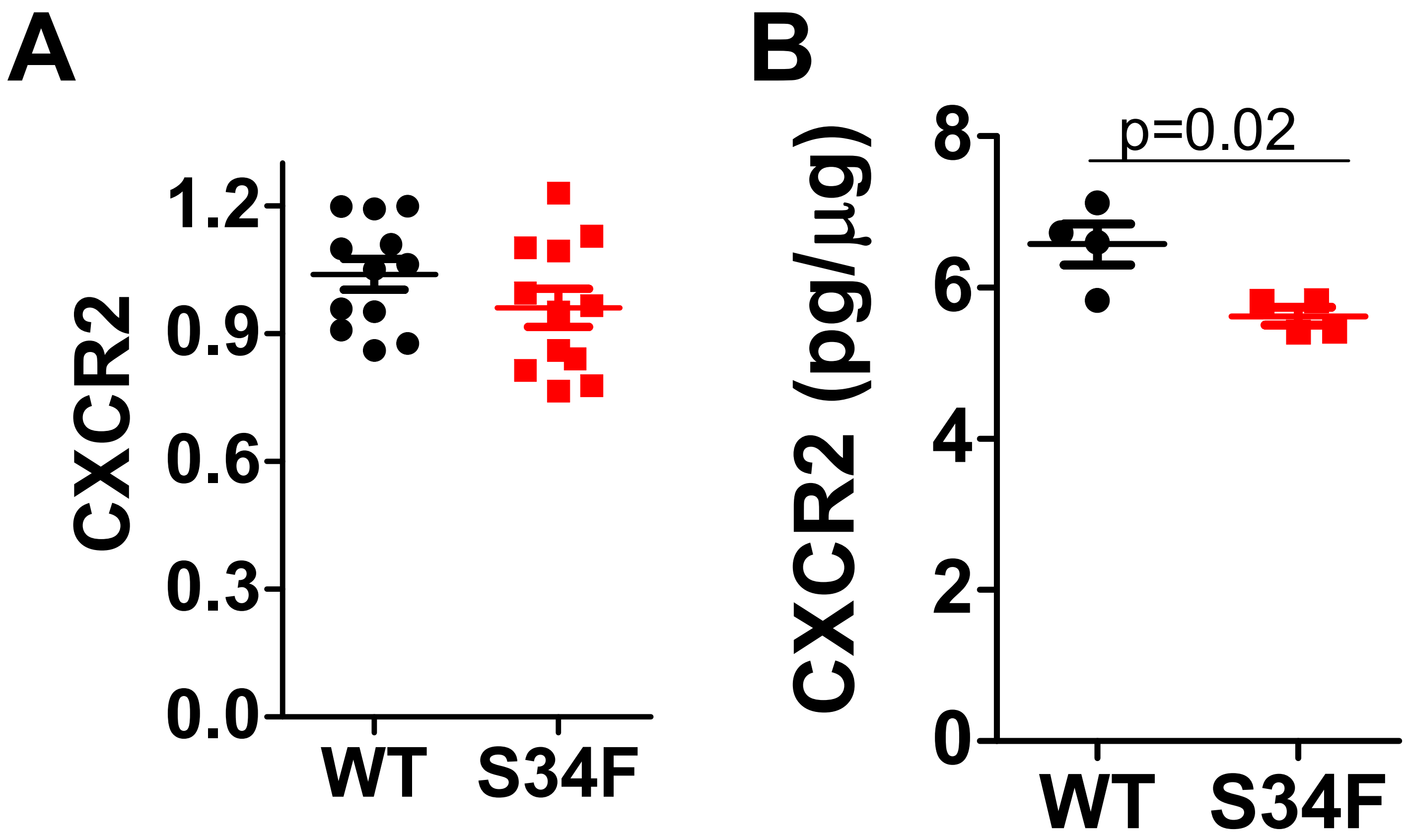

Supplementary Figure 3

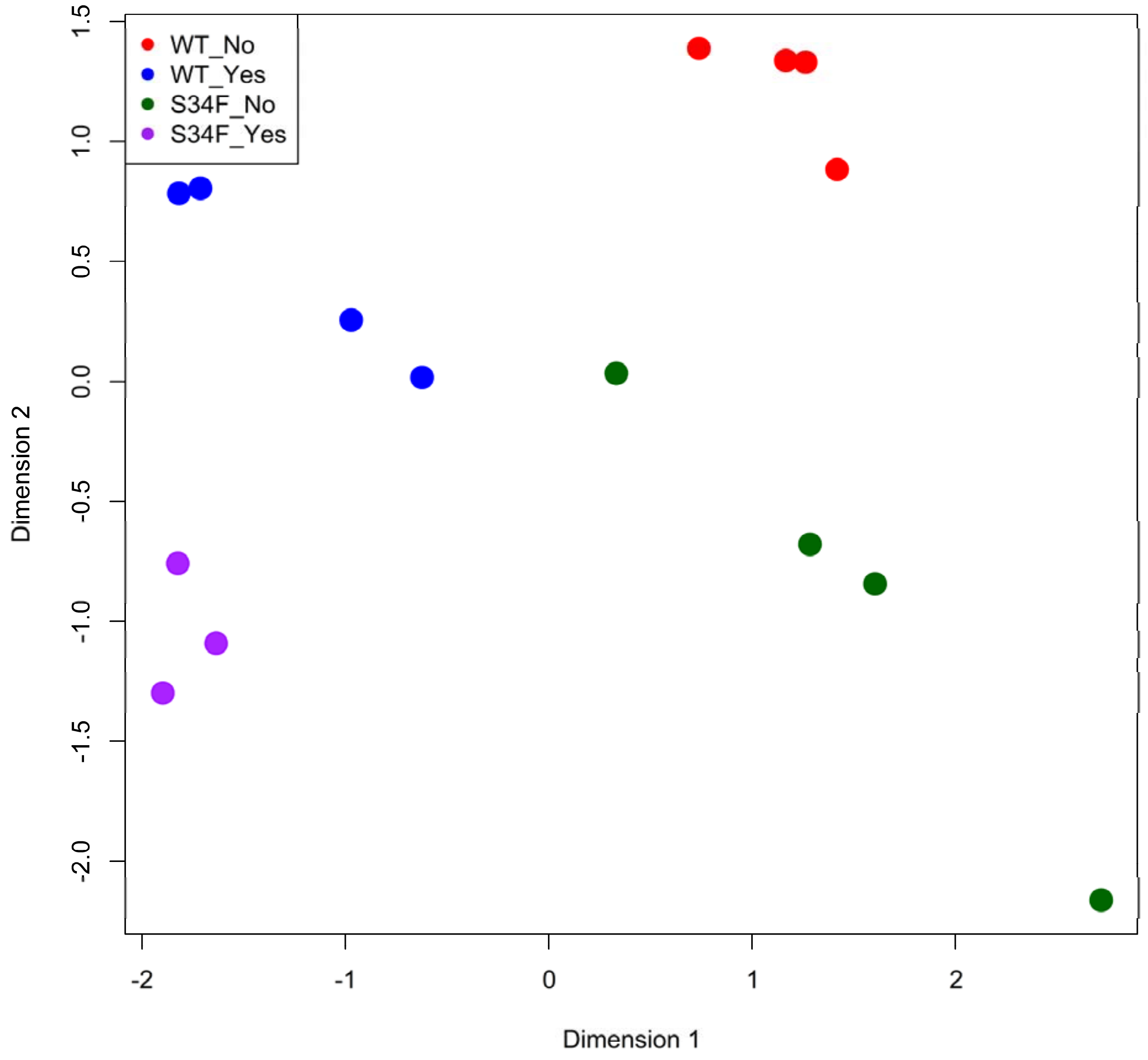

Supplementary Figure 4

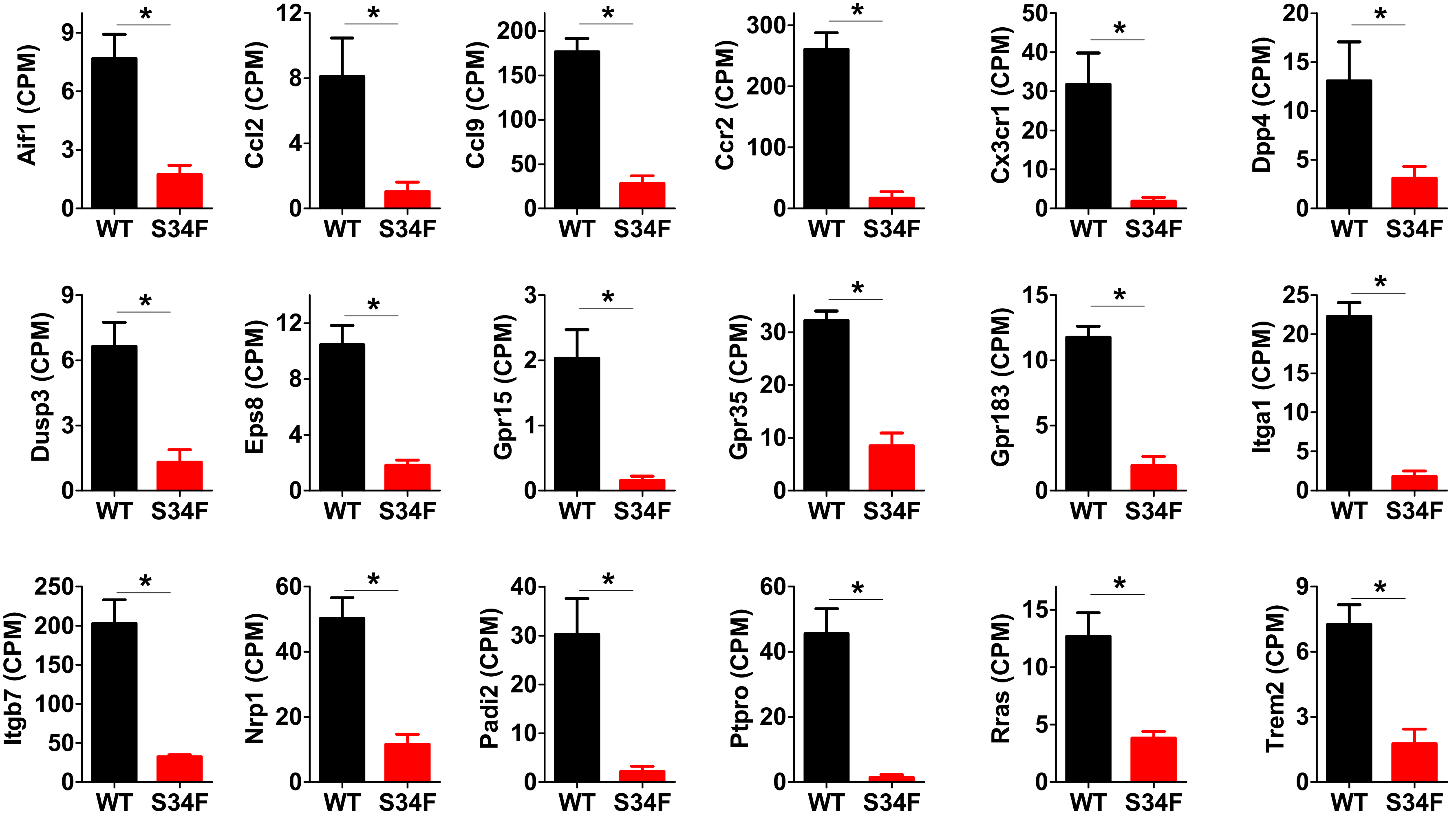

Supplementary Figure 5

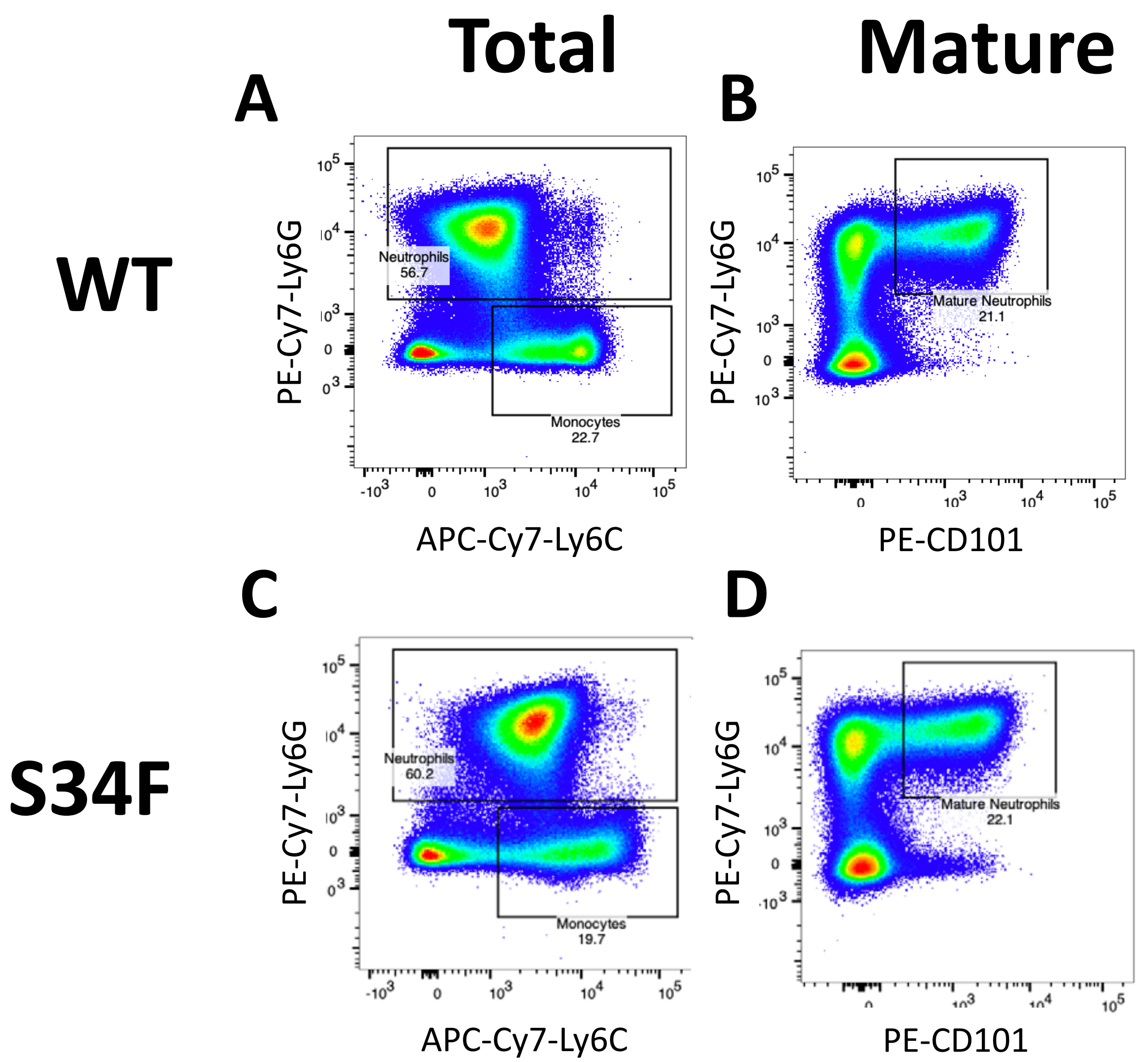

Supplementary Figure 6

Supplementary Table 1. MDS Patient Demographics

Age	Sex	Disease	% Blasts	Cytogenetics	IPSS-R	Spliceosome Mutation	Other Mutations
55	M	MDS-MLD	1	46,XY[18]	1.5	U2AF1-S34F	ASXL1
59	M	MDS-EB1	9	46,XY,dup(8)(q13q24)[6]/92,slx2[2]/46,XY[10]	5.5	U2AF1-S34F	BCOR, ETV6
82	M	MDS-MLD	2	46, XY	2.5	U2AF1-S34Y	TET2, MPL
67	F	MDS-RS-MLD	3.5	46,XX,add(10)(q24)[13]/46,XX[8]	4.5	SF3B1-K700Q	KIT, KRAS, TET2, CBL
69	M	MDS-RS	2	47,XY,+8[cp2]/46,XY[16]	2	SF3B1-K700E	None
68	F	MDS-RS-MLD	0	46,XX[20]	2	SF3B1-K700E	ASXL1
76	F	MDS-RS-MLD	3	46, XX	2.5	SF3B1-K700E	TET2
60	F	MDS-MLD	5	46,XX[19]	3.5	WT	RUNX1, STAG2
76	F	MDS-EB-2	12	45,XX,add(5)(q12),-7,-12,der(20)t(12;20)(q14;p11.2),+r[9]/44,sl,der(7) r(7)(p22q32)ins(7;?)(q32;?),der(11)ins(11;?)(q23;?)dup(11)(q21q23),+12 ,der(12;22)psudic(12;22)(p11.2;p11.2)ins(12;?)(p11.2;?),+20,der(20),-r[11]/46,XX[2].	8	WT	TP53, DNMT3A
71	F	MDS-MLD	1	45, XX, -7, del(12)(p12p13)[20]	3.5	WT	ASXL1, DNMT3A, SETBP1, TET2
63	M	MDS-MLD	3.5	46,XY,del(20)(q11.2q13.1)[20]	3	WT	ZRSR2 Gly251Trp
70	M	MDS-EB-2	12	46,XY,add(9)(q34),add(10)(q22)[cp14]/46,sl,del(7)(p16p13)[6]/46,sl,del (20)(q11.2q12)[2]/46,sdl1,del(20)[2]/46,XY[2]	8	WT	RUNX1, ASXL1
67	M	MDS-SLD	3	46, XY	2.5	WT	ASXL1, TET2, PHF6

Supplementary Table 2. Healthy blood donor demographics

Age(years)	Sex
63	Male
77	Male
70	Male
68	Male
73	Male
77	Male
74	Female
77	Male
64	Female
78	Female
70	Female
64	Female

Supplementary Table 5. Gene Ontology (GO) Analysis of genes undergoing alternative pre-mRNA splicing in U2AF1-S34F mice compared to U2AF1-wt mice (uninfected)

GO biological process complete	Fold enrichment	raw P-value	FDR
spliceosomal tri-snRNP complex assembly (GO:0000244)	6.45	4.03E-04	1.48E-02
error-prone translesion synthesis (GO:0042276)	5.65	1.60E-03	4.72E-02
mitotic recombination (GO:0006312)	4.29	1.30E-04	5.55E-03
DNA double-strand break processing (GO:0000729)	4.03	1.10E-03	3.47E-02
lactate metabolic process (GO:0006089)	3.99	6.69E-04	2.25E-02
positive regulation of translational initiation (GO:0045948)	3.99	6.69E-04	2.24E-02
microtubule nucleation (GO:0007020)	3.93	2.48E-04	9.83E-03
rRNA methylation (GO:0031167)	3.8	9.09E-04	2.92E-02
membrane protein ectodomain proteolysis (GO:0006509)	3.78	3.35E-04	1.27E-02
centriole replication (GO:0007099)	3.63	7.36E-04	2.44E-02
replication fork processing (GO:0031297)	3.63	5.44E-06	3.09E-04
centromere complex assembly (GO:0034508)	3.63	4.47E-04	1.62E-02
protein localization to microtubule organizing center (GO:1905508)	3.48	9.71E-04	3.10E-02
attachment of spindle microtubules to kinetochore (GO:0008608)	3.47	1.60E-03	4.71E-02
centrosome duplication (GO:0051298)	3.43	1.07E-04	4.71E-03
interstrand cross-link repair (GO:0036297)	3.4	2.86E-04	1.11E-02
centriole assembly (GO:0098534)	3.37	7.69E-04	2.53E-02
mitotic spindle assembly (GO:0090307)	3.34	9.76E-06	5.27E-04
DNA-templated DNA replication maintenance of fidelity (GO:0045005)	3.27	1.26E-05	6.68E-04

DNA synthesis involved in DNA repair (GO:0000731)	3.23	2.91E-04	1.12E-02
TOR signaling (GO:0031929)	3.16	2.28E-04	9.18E-03
positive regulation of double-strand break repair via homologous recombination (GO:1905168)	3.16	2.28E-04	9.16E-03
DNA-templated DNA replication (GO:0006261)	3.13	1.47E-10	1.59E-08
post-transcriptional gene silencing (GO:0016441)	3.11	6.02E-04	2.05E-02
bone cell development (GO:0098751)	3.08	9.81E-04	3.12E-02

Supplementary Table 6. Gene Ontology (GO) Analysis of genes upregulated in neutrophils from U2AF1-S34F mice compared to U2AF1-wt mice (uninfected)

GO biological process complete	Fold enrichment	raw P-value	FDR
synapse pruning (GO:0098883)	67.34	2.34E-05	9.72E-03
cell junction disassembly (GO:0150146)	56.11	3.70E-05	1.36E-02
regulation of ribonuclease activity (GO:0060700)	44.89	6.58E-05	2.16E-02
positive regulation of monocyte chemotactic protein-1 production (GO:0071639)	35.44	1.23E-04	3.33E-02
cellular response to interferon-alpha (GO:0035457)	35.44	1.23E-04	3.27E-02
peptide cross-linking (GO:0018149)	21.38	5.15E-05	1.76E-02
positive regulation of cholesterol transport (GO:0032376)	19.52	7.17E-05	2.26E-02
positive regulation of sterol transport (GO:0032373)	19.52	7.17E-05	2.21E-02
regulation of plasma lipoprotein particle levels (GO:0097006)	17.6	1.04E-04	2.94E-02
cellular response to interferon-beta (GO:0035458)	16.03	1.47E-04	3.79E-02
complement activation, classical pathway (GO:0006958)	11.59	4.70E-09	7.41E-06
humoral immune response mediated by circulating immunoglobulin (GO:0002455)	11.38	5.66E-09	7.43E-06
positive regulation of lipid localization (GO:1905954)	10.86	2.50E-05	9.84E-03
complement activation (GO:0006956)	10.69	1.05E-08	1.18E-05
regulation of lipid localization (GO:1905952)	8.45	2.46E-05	9.95E-03
immunoglobulin mediated immune response (GO:0016064)	8.43	1.08E-07	9.47E-05
B cell mediated immunity (GO:0019724)	8.31	1.23E-07	9.72E-05
phagocytosis, recognition (GO:0006910)	7.74	4.22E-05	1.48E-02

phagocytosis, engulfment (GO:0006911)	7.67	1.24E-05	6.32E-03
defense response to virus (GO:0051607)	7.67	1.24E-05	6.12E-03
defense response to symbiont (GO:0140546)	7.64	1.28E-05	6.12E-03
plasma membrane invagination (GO:0099024)	7.39	1.62E-05	7.29E-03
membrane invagination (GO:0010324)	7.18	1.97E-05	8.40E-03
B cell receptor signaling pathway (GO:0050853)	7.08	7.29E-05	2.21E-02
lymphocyte mediated immunity (GO:0002449)	6.66	1.04E-06	7.15E-04

Supplementary Table 7. Gene Ontology (GO) Analysis of genes upregulated in neutrophils from U2AF1-S34F mice compared to U2AF1-wt mice (infected)

GO biological process complete	Fold enrichment	raw P-value	FDR
antigen processing and presentation of exogenous peptide antigen via MHC class I, TAP-dependent (GO:0002479)	> 100	5.42E-04	4.43E-02
ISG15-protein conjugation (GO:0032020)	61.96	4.70E-05	5.88E-03
positive regulation of interferon-gamma-mediated signaling pathway (GO:0060335)	59.01	2.57E-06	4.77E-04
positive regulation of response to interferon-gamma (GO:0060332)	59.01	2.57E-06	4.72E-04
regulation of interferon-gamma-mediated signaling pathway (GO:0060334)	41.31	3.49E-08	9.17E-06
regulation of response to interferon-gamma (GO:0060330)	41.31	3.49E-08	9.02E-06
positive regulation of RIG-I signaling pathway (GO:1900246)	41.31	7.63E-06	1.27E-03
protein mono-ADP-ribosylation (GO:0140289)	34.42	1.37E-05	2.13E-03
protein poly-ADP-ribosylation (GO:0070212)	34.42	1.79E-04	1.84E-02
nucleotide-binding oligomerization domain containing 2 signaling pathway (GO:0070431)	30.98	2.32E-04	2.28E-02
nucleotide-binding oligomerization domain containing signaling pathway (GO:0070423)	30.98	2.32E-04	2.27E-02
cytoplasmic pattern recognition receptor signaling pathway in response to virus (GO:0039528)	30.98	2.32E-04	2.25E-02
adhesion of symbiont to host (GO:0044406)	29.51	2.26E-05	3.22E-03
cellular response to interferon-beta (GO:0035458)	29.51	7.43E-18	6.17E-15
response to interferon-beta (GO:0035456)	29.05	7.76E-20	7.65E-17
nucleotide-binding domain, leucine rich repeat containing receptor signaling pathway (GO:0035872)	28.17	2.93E-04	2.72E-02
cytoplasmic pattern recognition receptor signaling pathway (GO:0002753)	28.17	2.29E-07	5.01E-05
cellular response to interferon-alpha (GO:0035457)	27.18	2.84E-06	5.14E-04

response to interferon-alpha (GO:0035455)	26.65	2.99E-09	9.82E-07
regulation of protein complex stability (GO:0061635)	25.82	3.63E-04	3.25E-02
antigen processing and presentation of exogenous peptide antigen via MHC class I (GO:0042590)	23.83	4.44E-04	3.78E-02
negative regulation of viral genome replication (GO:0045071)	23.15	1.43E-13	8.06E-11
regulation of RIG-I signaling pathway (GO:0039535)	22.95	5.25E-05	6.47E-03
positive regulation of type I interferon-mediated signaling pathway (GO:0060340)	21.74	6.31E-05	7.59E-03
positive regulation of pattern recognition receptor signaling pathway (GO:0062208)	20.65	2.12E-09	7.43E-07

Supplementary Table 8. Gene Ontology (GO) Analysis of genes downregulated in neutrophils from U2AF1-S34F mice compared to U2AF1-wt mice (uninfected)

GO biological process complete	Fold enrichment	raw P-value	FDR
positive regulation of neuroinflammatory response (GO:0150078)	25.37	4.35E-04	4.54E-02
antigen processing and presentation of exogenous peptide antigen via MHC class I (GO:0042590)	23.42	5.77E-05	1.07E-02
ceramide catabolic process (GO:0046514)	15.22	2.40E-04	2.98E-02
regulation of glial cell migration (GO:1903975)	15.22	3.87E-05	8.03E-03
positive regulation of monocyte chemotaxis (GO:0090026)	13.84	3.31E-04	3.70E-02
T cell migration (GO:0072678)	12.69	8.36E-05	1.39E-02
antigen processing and presentation of exogenous peptide antigen (GO:0002478)	12.34	1.84E-05	4.75E-03
protein activation cascade (GO:0072376)	12.18	5.09E-04	4.90E-02
positive regulation of interleukin-1 beta production (GO:0032731)	10.87	3.81E-07	2.23E-04
antigen processing and presentation of exogenous antigen (GO:0019884)	10.38	4.44E-05	8.75E-03
mature B cell differentiation (GO:0002335)	10.29	2.03E-04	2.65E-02
activation of innate immune response (GO:0002218)	10.15	2.73E-06	9.37E-04
positive regulation of interleukin-1 production (GO:0032732)	9.13	1.44E-06	5.28E-04
regulation of interleukin-1 beta production (GO:0032651)	8.63	1.63E-07	1.23E-04
response to interferon-beta (GO:0035456)	8.32	3.75E-05	7.99E-03
cellular extravasation (GO:0045123)	8.27	5.11E-04	4.88E-02
cellular response to interferon-beta (GO:0035458)	8.16	1.51E-04	2.16E-02
chemokine-mediated signaling pathway (GO:0070098)	7.87	1.80E-04	2.41E-02

negative regulation of chemotaxis (GO:0050922)	7.61	2.14E-04	2.72E-02
regulation of interleukin-1 production (GO:0032652)	7.1	9.90E-07	4.46E-04
cellular response to chemokine (GO:1990869)	6.72	3.99E-04	4.25E-02
response to chemokine (GO:1990868)	6.72	3.99E-04	4.23E-02
lymphocyte migration (GO:0072676)	6.52	4.61E-04	4.60E-02
hemostasis (GO:0007599)	6.34	7.80E-06	2.28E-03
mononuclear cell migration (GO:0071674)	6.28	6.82E-05	1.21E-02

Supplementary Table 9. Gene Ontology (GO) Analysis of genes downregulated in neutrophils from U2AF1-S34F mice compared to U2AF1-wt mice (infected)

GO biological process complete	Fold enrichment	raw P-value	FDR
ceramide catabolic process (GO:0046514)	20.75	7.38E-05	1.94E-02
ceramide metabolic process (GO:0006672)	9.93	6.42E-07	1.01E-03
positive regulation of phagocytosis (GO:0050766)	9.22	4.55E-06	4.22E-03
sphingolipid metabolic process (GO:0006665)	8.04	2.64E-07	5.95E-04
regulation of phagocytosis (GO:0050764)	7.65	4.80E-06	3.98E-03
mononuclear cell migration (GO:0071674)	7.49	6.35E-05	1.79E-02
membrane lipid metabolic process (GO:0006643)	6.77	4.29E-07	8.45E-04
regulation of interleukin-6 production (GO:0032675)	5.63	4.87E-05	1.51E-02
positive regulation of lymphocyte proliferation (GO:0050671)	5.57	1.37E-04	3.32E-02
positive regulation of mononuclear cell proliferation (GO:0032946)	5.46	1.56E-04	3.72E-02
leukocyte migration (GO:0050900)	4.76	3.14E-05	1.15E-02
regulation of production of molecular mediator of immune response (GO:0002700)	4.62	2.03E-04	4.50E-02
positive regulation of T cell activation (GO:0050870)	4.62	4.03E-05	1.38E-02
positive regulation of leukocyte cell-cell adhesion (GO:1903039)	4.56	2.04E-05	8.91E-03
cytokine-mediated signaling pathway (GO:0019221)	4.26	1.86E-05	8.61E-03
regulation of leukocyte cell-cell adhesion (GO:1903037)	4.17	5.26E-06	3.95E-03
positive regulation of cell-cell adhesion (GO:0022409)	4.15	2.39E-05	1.02E-02

regulation of lymphocyte proliferation (GO:0050670)	4.12	2.23E-04	4.81E-02
regulation of inflammatory response (GO:0050727)	4	1.71E-05	8.42E-03
positive regulation of cytokine production (GO:0001819)	3.9	7.40E-07	1.06E-03
negative regulation of cytokine production (GO:0001818)	3.87	1.83E-04	4.13E-02
regulation of cytokine production (GO:0001817)	3.86	9.24E-10	1.46E-05
regulation of T cell activation (GO:0050863)	3.84	2.63E-05	1.01E-02
positive regulation of defense response (GO:0031349)	3.77	2.29E-04	4.81E-02
regulation of cell-cell adhesion (GO:0022407)	3.74	2.60E-06	2.73E-03

Supplementary Table 10. Genes that are downregulated in neutrophils from U2AF1-S34F mice in selected GO categories related to neutrophil migration

Gene	LogFC	1	2	3	4	5	6	7	8	9	10	Oligo 1	Oligo 2
Ptpro	-5.15866										X	ACC CAA ACA GCA CAG AAC TGC CCC TTA TCC	CCC AGT TGT TTT TGT TCA AGT TTA CGA TAC GAA C
Gpr15	-4.30449			X						X	X	GCC TGC TGG GGT TGC CTA CTC TTC TGT CCA G	CTT TTT CTC TGC ACA GTA TGG CTT ATC ATC AAT CAG
Cx3cr1	-4.10387	X	X		X	X		X	X			CTG GGT GGA GAA TGG CCA GTG GGG CCT TCA C	TCG TAC TCA AAG TTT TCT GTC ACT GAT TCA GGG AAC
Ccr2	-4.08015	X	X	X	X	X		X	X	X	X	GCC TTC GTT GGG GAG AAG TTC AGA AGC CTT TTT CAC	CTG GTT TTT GGA GTG GGG CAA TCC TAC AGC C
Padi2	-3.89743						X					GTG GTG GAG AAG AAC AAC CCA AAG AAG GCA TCC TGG	ACC AGC AGG ATG GCC CCC TGG CCC TCG G
Itga1	-3.71072				X							GTC ACC AAC CCA AAT GGA GGA TTT CTG GCT TGT GG	TCC AGT TGT GTA ATG CAA ATG TCC ACA TCT ATA G
Ccl2	-3.10049		X	X	X	X	X	X	X	X	X	Too low to detect in human cells	
Gpr183	-2.70375	X		X						X	X	Too low to detect in human cells	
Ccl9	-2.67907					X		X	X	X	X	No human ortholog	
Itgb7	-2.56809			X	X					X	X	GGT CCG GGT CAC GCT GCG GCC TGG GGA GCC	GGT CCA CCG GGT ATC CCT CAG CAC GAA GGA AGC
Eps8	-2.45829										X	Too low to detect in human cells	
Dusp3	-2.42891						X					Too low to detect in human cells	
Aif1	-2.21685		X				X					GTT TGA CCT TAA TGG AAA TGG CGA TAT TGA TAT CAT G	TCT TGG GGA CTC CAA GTT TCT CCA GCA TTC G
Dpp4	-2.16204						X					Not tested	
Trem2	-2.14846	X				X		X	X			Not tested	
Nrp1	-2.06053						X					Not tested	
Gpr35	-1.82709					X		X	X			Not tested	
Rras	-1.71862	X										Not tested	

Gene refers to the official gene symbol. LogFC is the log(2-fold change) for expression of the mouse gene in neutrophils in U2AF1-S34F mice compared to U2AF1-wt mice. 1-10 indicates that that gene exhibits decreased expression in the indicated GO categories listed below. Oligos 1 and 2 are the oligonucleotides used to monitor gene expression of the human orthologs in human neutrophils using qPCR.

[1]regulation of glial cell migration (GO:1903975)
[2]positive regulation of monocyte chemotaxis (GO:0090026)
[3]T cell migration (GO:0072678)
[4]cellular extravasation (GO:0045123)
[5]chemokine-mediated signaling pathway (GO:0070098)

[6]negative regulation of chemotaxis (GO:0050922)
[7]cellular response to chemokine (GO:1990869)
[8]response to chemokine (GO:1990868)
[9]lymphocyte migration (GO:0072676)
[10]mononuclear cell migration (GO:0071674)

Supplementary Table 11. Ingenuity Pathway Analysis (IPA) of gene expression changes in neutrophils induced by U2AF1-S34F in the absence of infection

Categories	Diseases or Function Annotation	p-value	Predicted Activation State	Activation z-score	# of Molecules
Immunological Disease,Inflammatory Disease	Immune mediated inflammatory disease	6.5E-22		-1.385	105
Cellular Movement,Hematological System Development and Function,Immune Cell Trafficking	Cell movement of leukocytes	7.92E-22	Decreased	-2.122	79
Cell-To-Cell Signaling and Interaction,Hematological System Development and Function,Immune Cell Trafficking,Inflammatory Response	Activation of leukocytes	2.41E-21	Decreased	-2.42	71
Cellular Movement,Immune Cell Trafficking	Leukocyte migration	2.49E-21	Decreased	-2.284	86
Cell-To-Cell Signaling and Interaction,Hematological System Development and Function	Activation of blood cells	8.04E-21	Decreased	-2.357	74
Immunological Disease	Systemic autoimmune syndrome	2.22E-20		-1.539	101
Hematological System Development and Function,Tissue Morphology	Quantity of leukocytes	3.16E-20	Decreased	-4.166	83
Cell-To-Cell Signaling and Interaction	Activation of cells	3.43E-20	Decreased	-2.658	86
Cellular Movement	Migration of cells	7.08E-20	Decreased	-3.568	138
Hematological System Development and Function,Tissue Morphology	Quantity of blood cells	7.35E-20	Decreased	-3.815	88
Cellular Movement,Hematological System Development and Function,Immune Cell Trafficking,Inflammatory Response	Cell movement of phagocytes	2.88E-19	Decreased	-2.504	62
Inflammatory Response	Inflammation of absolute anatomical region	5.28E-19		-0.253	96
Cell Death and Survival	Apoptosis	6.74E-19		1.442	154

Inflammatory Response,Organismal Injury and Abnormalities	Inflammation of organ	1.52E-18		-0.42	105
Cellular Growth and Proliferation,Lymphoid Tissue Structure and Development	Proliferation of lymphatic system cells	3.02E-18	Decreased	-2.679	70
Endocrine System Disorders,Gastrointestinal Disease,Immunological Disease,Metabolic Disease,Organismal Injury and Abnormalities	Insulin-dependent diabetes mellitus	3.75E-18		-1.111	49
Cellular Movement,Hematological System Development and Function,Immune Cell Trafficking	Cell movement of antigen presenting cells	5.22E-18	Decreased	-2.088	47
Cellular Movement	Cell movement	6.39E-18	Decreased	-3.618	143
Cancer,Organismal Injury and Abnormalities	Formation of solid tumor	7.44E-18		-1.442	355
Cellular Development,Cellular Growth and Proliferation,Hematological System Development and Function,Lymphoid Tissue Structure and Development	Proliferation of immune cells	8.28E-18	Decreased	-2.676	69
Lymphoid Tissue Structure and Development,Tissue Morphology	Quantity of lymphatic system cells	1.21E-17	Decreased	-3.28	70
Cellular Function and Maintenance	Function of blood cells	1.61E-17		-0.896	52
Tissue Morphology	Quantity of cells	1.96E-17	Decreased	-3.879	121
Cellular Function and Maintenance	Function of leukocytes	2.14E-17		-0.896	49
Cellular Development,Cellular Growth and Proliferation,Hematological System Development and Function,Lymphoid Tissue Structure and Development	Proliferation of mononuclear leukocytes	2.49E-17	Decreased	-2.419	66

Cell migration-relevant pathways highlighted in yellow.
